# Targeting calcineurin: a synergistic strategy with caspofungin to induce farnesol-mediated quorum sensing in *Nakaseomyces glabrata*—an inoculum-dependent tale

**DOI:** 10.1042/BCJ20253193

**Published:** 2025-12-24

**Authors:** Yu-Ke Cen, Lu-Lu Zhang, Zi-Jie Zhang, Yu-Jie Zhang, Tao-Xu Lu, Wan-Ying Zhou, Chao Xiang, Ya-Ping Xue, Yu-Guo Zheng

**Affiliations:** 1 Key Laboratory of Bioorganic Synthesis of Zhejiang Province, College of Biotechnology and Bioengineering, Zhejiang University of Technology, Hangzhou, 310014, China; 2 Engineering Research Center of Bioconversion and Biopurification of Ministry of Education, Zhejiang University of Technology, Hangzhou, 310014, China; 3 National and Local Joint Engineering Research Center for Biomanufacturing of Chiral Chemicals, Zhejiang University of Technology, Hangzhou, 310014, China

**Keywords:** biofilms, calcineurin, caspofungin, cell wall, *Nakaseomyces glabrata*, quorum sensing

## Abstract

Using *Nakaseomyces glabrata* as a model organism, we demonstrate that targeting calcineurin can synergize with caspofungin to induce a quorum sensing (QS) effect mediated by farnesol. This QS effect requires calcineurin deficiency, sub-minimum inhibitory concentration (MIC) levels of caspofungin, and a high-density cell population. Cell growth and biofilm formation were significantly inhibited within a specific range of cell density and sub-MIC caspofungin treatment in the calcineurin mutant. The inhibition of biofilm formation follows the ‘paradoxical growth,’ showing a concentration-dependent response to caspofungin. We show that high cell density triggers two antagonistic effects: overcoming antibiotic inhibition, which promotes cell propagation, and QS-mediated growth inhibition, which negatively regulates cell proliferation. The QS molecule farnesol was detectable only in the calcineurin mutant, where the transcription of the farnesol synthase Dpp3 was significantly up-regulated, and deletion of *DPP3* abolished the QS effect in both spot assay and biofilm formation of the calcineurin mutant. Besides this, we identified a Dpp3-dependent, ergosterol-farnesol metabolism-linked Crz1-independent regulatory mechanism that contributes to the calcineurin-mediated multi-stress resistance. We demonstrate that calcineurin, Dpp3, and caspofungin are all involved in regulating ergosterol metabolism and the transcription of *ERG11* and *FKS* genes, leading to significant changes in membrane and cell wall stress tolerance. The cell wall composition undergoes substantial alterations upon deletion of calcineurin or treatment with caspofungin, while caspofungin also increases the levels of β-glucan and short peptides in the medium, tentatively pointing to the release of QS inducers from the cell wall.

## Introduction

Fungal infections have emerged as a significant global public health issue, with invasive cases rising sharply and high mortality rates [1,2]. *Candida* species are the most prevalent fungal pathogens, with non-*albicans Candida* demonstrating notable intrinsic and acquired antifungal resistance [3]. A prominent example is *Nakaseomyces glabrata* (previously known as *Candida glabrata*), which is the second leading cause of invasive candidiasis worldwide, excluding Latin America, and has been classified as a high-priority pathogen by the World Health Organization (WHO) [4].

Calcineurin has been widely considered a promising antifungal target, particularly when used in combination with other antifungals, whereas very little is known about the underlying mechanisms [5,6]. This serine/threonine-specific protein phosphatase is composed of a regulatory subunit (Cnb1) and a catalytic subunit (Cna1) and loses its activity when either subunit is knocked out. The hallmark downstream target of calcineurin is the transcription factor Crz1, which regulates the transcription of hundreds of genes, many of which are involved in resistance against different stresses [7,8]. Calcineurin-Crz1 also regulates resistance against azoles and polyenes. However, the calcineurin and Crz1 mutants exhibit distinct effects in this regulation. For instance, compared with the wildtype (WT) strain, the calcineurin mutant exhibits greater sensitivity to fluconazole, whereas the Crz1 mutant demonstrates increased resistance. These differences also correlate with divergent ergosterol accumulation [9], suggesting that calcineurin regulates the mevalonate–ergosterol pathway through mechanisms beyond Crz1. Calcineurin is also crucial for cell wall biosynthesis in *N. glabrata* [10]*.* Genome-wide transcriptomic analysis has revealed that calcineurin up-regulates the expression of proteins related to cell wall biosynthesis and participates in cell wall remodeling by regulating the glycosylation level [11]. On the other hand, echinocandins are the cell wall-targeting antifungals that are commonly used as a first-line treatment for fungal infections. As a representative, caspofungin, the first FDA-approved agent in this class, exerts its antifungal activity by non-competitive inhibition of β-(1,3)-glucan synthase (Fks) [12,13]. This mechanism disrupts fungal cell wall integrity through β-glucan depletion, rendering *Candida* cells vulnerable to osmotic stress and ultimately leading to cell lysis [14].

Quorum sensing (QS) is a cell density-dependent signaling mechanism that co-ordinates microbial group behaviors such as virulence factor production, biofilm formation, and antibiotic resistance [15]. Taking biofilm as an example, the extracellular matrix directly impedes antibiotic penetration, while co-ordinated cell density-dependent behaviors, enabled by QS effects, drive metabolic specialization and virulence expression [16]. This defense-adaptation framework underscores the therapeutic challenges. When the microbial population density reaches a certain threshold, cells may secrete and accumulate diffusible signaling quorum sensing molecules (QSMs) to adapt to environmental changes [17]. Farnesol is the first QSM identified in *Candida* and plays multiple roles, including inhibiting hyphal growth, modulating biofilm formation, and inducing apoptosis [18–20]. However, very little is known about the mechanisms governing the metabolism, induction, and regulation of QSMs.

**Figure 1:**
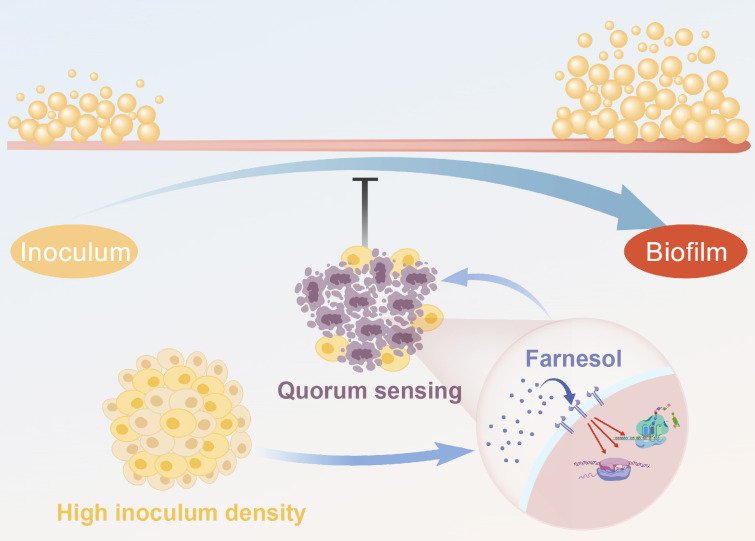
Schematic diagram illustrating the farnesol-mediated quorum sensing in the regulation of biofilm formation. High inoculum density may induce the production of the QS molecule farnesol, and thus inhibit biofilm formation. The schematic diagram is drawn by BioGDP [21].

We observed a QS-like effect in *N. glabrata* when the calcineurin mutant was treated with sub-minimum inhibitory concentration (MIC) caspofungin. The growth of the calcineurin mutant, as measured by both spot assays and biofilm formation, was inhibited in an inoculum cell density-dependent manner. This effect was abolished upon deletion of *DPP3*, the gene encoding the putative phosphatase catalyzing the conversion of farnesol diphosphate to farnesol. Notably, during biofilm formation, increased inoculum density triggers the QS effect, while the increasing cell population may also counteract the antibiotic’s inhibitory effects. We also observed a typical echinocandin-specific ‘paradoxical growth’ that the QS effect displayed in a caspofungin-concentration dependent way. This QS effect was not observed during the biofilm formation of the calcineurin-*DPP3* double mutant. Besides this, deletion of *DPP3* also restored mature biofilm formation by the calcineurin mutant without caspofungin added. Figure 1 demonstrates the role of farnesol-mediated QS in the regulation of biofilm formation.

Moreover, deletion of *DPP3* restored the calcineurin mutant’s tolerance to cell wall stress and increased ergosterol accumulation. Compared with the WT strain, the calcineurin mutant exhibits significantly higher *DPP3* transcription, which aligns with the observation that farnesol is detectable in the calcineurin mutant but not in the WT strain. On the other hand, transcription of *ERG11* is up-regulated by either deletion of calcineurin, deletion of *DPP3*, or the addition of caspofungin, partially explaining the restored resistance to fluconazole and sodium dodecyl sulfate (SDS) observed when *DPP3* was deleted from the calcineurin mutant. We also demonstrate that calcineurin deletion and caspofungin treatment significantly alter cell wall composition, raising the intriguing possibility that cell wall damage might release certain molecules capable of modulating QS.

In this study, we aimed to elucidate the mechanism by which caspofungin and high cell density induce the QS response in a calcineurin-deficient background. Three interconnected cellular processes were identified as potentially influencing cell fitness and growth: compromised cell wall integrity, altered ergosterol-related membrane stability, and other stress resistance mechanisms. First, caspofungin-induced cell wall damage, synergizing with calcineurin deletion in affecting Fks function, led us to hypothesize that cell wall-derived components probably act as direct QS inducers. Second, calcineurin-dependent ergosterol synthesis, which correlates with DPP3-mediated farnesol production, appears to influence membrane integrity. Finally, we demonstrated Dpp3-dependent stress resistance connected to ergosterol-farnesol metabolism, a pathway independent of Crz1, despite Crz1’s known involvement in other stress responses. Together, these components constitute a co-ordinated network that maintains cellular fitness under stress conditions.

## Results

### Caspofungin triggers quorum sensing effect in calcineurin mutant

**Figure 2:**
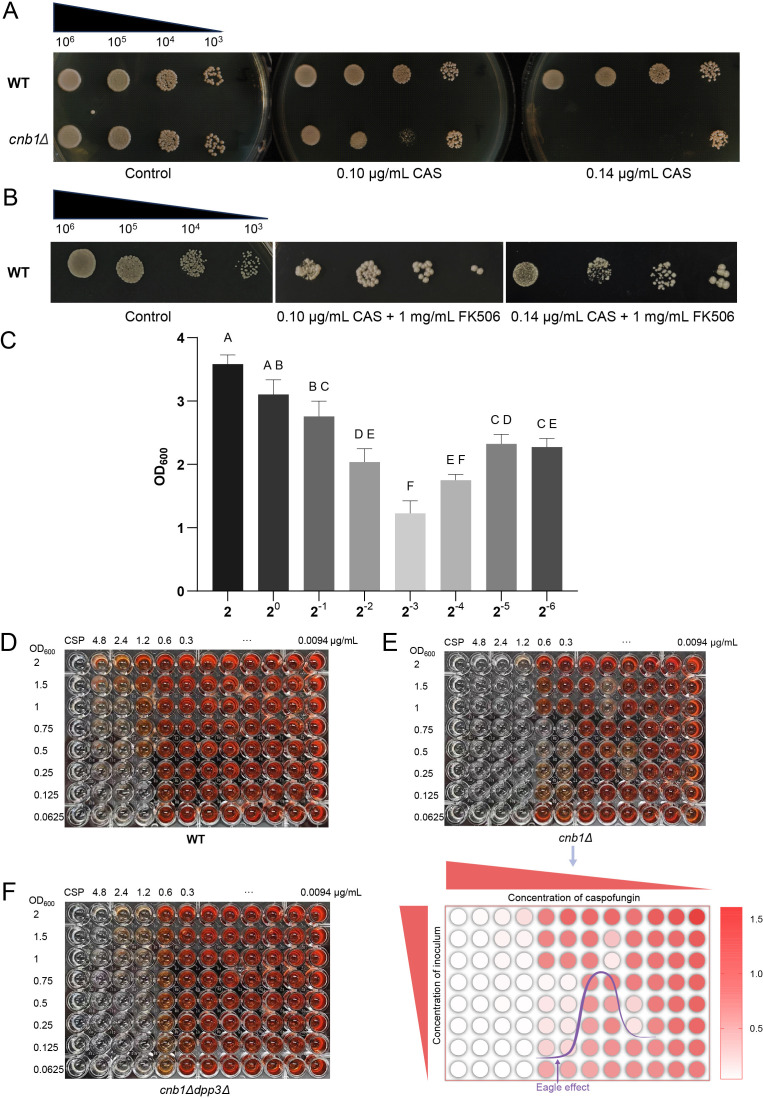
Quorum sensing effects induced by calcineurin deletion and caspofungin treatment. **(A)** Spot assay of wildtype (WT) and *cnb1Δ* strains treated with 0.01 µg/ml or 0.014 µg/ml caspofungin. (**B)** Spot assay of the WT strain treated with a combination of caspofungin (0.1 µg/ml and 0.14 µg/ml) and tacrolimus (FK506, 1 mg/ml). (**C)** Induction of quorum sensing by FK506 and caspofungin. OD_600_ was measured after 24 h of growth at 37°C. X axis: two-fold serial dilution of cells with the highest OD_600_ of 2 (final OD_600_ =2, 1, 0.5, 0.25, 0.125, 0.0625, 0.03125, 0.015625, and 0.0078 in a 200 µl total volume). Capital letters (**A, B, C, D, E, F**) above the data bars indicate statistical significance: bars sharing the same letter denote *P*>0.05, while bars with different letters denote *P* <0.05. Inoculum-gradient and caspofungin-gradient biofilm formation assays of (**D)** wildtype (plate: WT), (**E)** calcineurin mutant (plate: *cnb1Δ*), and (**F)** *cnb1Δdpp3Δ* strains (plate: *cnb1Δdpp3Δ*). Biofilm formation was assessed using a range of inoculum concentrations (from bottom to top), with OD_600_ values adjusted from 0.0625 (bottom row) to 2 (top row). Caspofungin was added (in a range of concentrations from left to right) after the adhesion phase in fresh medium, using two-fold serial dilutions to achieve final concentrations ranging from 0.009375 µg/ml (right column) to 4.8 µg/ml (left column). Biofilm was quantified using the XTT assay. Metabolically active cells reduce the yellow XTT salt to an orange-red, water-soluble formazan product. The intensity of the resulting color is directly proportional to the metabolic activity and thus the amount of viable biomass in the biofilm. CAS: caspofungin. WT: the wild type strain.

A QS-like effect was observed when the calcineurin mutant *cnb1Δ* was treated with a sub-MIC concentration of caspofungin in a spot assay (see Supplementary Figure S1 for MIC test). When 0.14 µg/ml caspofungin was used, the growth of the high-concentration inoculum spot was inhibited, except for the lowest-concentration inoculum spot, which exhibited WT-like growth (Figure 2A). This effect was not observed previously in the *crz1Δ* strain [9]. When caspofungin was added at a lower concentration of 0.1 µg/ml, the inhibited growth of the high-concentration inoculum spot was overcome, while the sub-high-concentration inoculum spot remained inhibited. We also tested this QS-like effect using the calcineurin inhibitor FK506 (tacrolimus), which may induce a similar QS effect in the WT strain (Figure 2B). To quantitatively assess the QS effect under co-treatment with FK506 and caspofungin, the inoculum was subjected to two-fold serial dilution in a microtiter plate. The results revealed that growth was significantly reduced in wells with three intermediate inoculum concentrations compared with those containing either higher or lower inoculum levels (Figure 2C). This result is consistent with the spot assay findings using the calcineurin mutant or the addition of FK506 to the WT strain. Since the QSM farnesol is on the metabolic shunt of the mevalonate–ergosterol pathway, and calcineurin influences ergosterol metabolism, we determined the farnesol content in the medium. The results indicated that farnesol can be determined in the *cnb1Δ* strain (Supplementary Figure S2), whereas its levels in the WT strain were below the gas chromatography-mass spectrometry (GC-MS) detection limit. Biofilm formation is a critical virulence factor in *N. glabrata*, and its high cell density property meets the necessity of triggering QS effects. Thus, we conducted an *in vitro* biofilm assay using progressively increasing inoculum cell densities and escalating concentrations of caspofungin. The results demonstrated that with the calcineurin mutant, not the WT strain (Figure 2D), biofilm formation transitioned from well-established to inhibited as the inoculum density increased at a fixed concentration of caspofungin (Figure 2E: in each column, the cell density of the inoculum increases from bottom to top). However, this inhibition was eventually overcome at even higher cell densities, leading to the restoration of biofilm formation. Increased cell inoculum density typically enhances biofilm formation. Our result strongly supports the QS effect observed in the spot assay. The increased inoculum density triggered the QS effect, while the growing cell population may have also mitigated the antibiotic’s inhibitory effects. As a result, when the inoculum density reached a sufficiently high level, the QS-induced growth inhibition was retrieved due to the consumption/overcoming of caspofungin by the cell population. Interestingly, this biofilm inhibition initially required an increase in cell inoculum density, followed by a decrease as the concentration of caspofungin was raised. This result supports the ‘paradoxical growth’ of echinocandins and further strengthens the antifungal-QS dual effect [22].

### The putative farnesol synthase *DPP3* is essential for the quorum sensing effect

**Figure 3:**
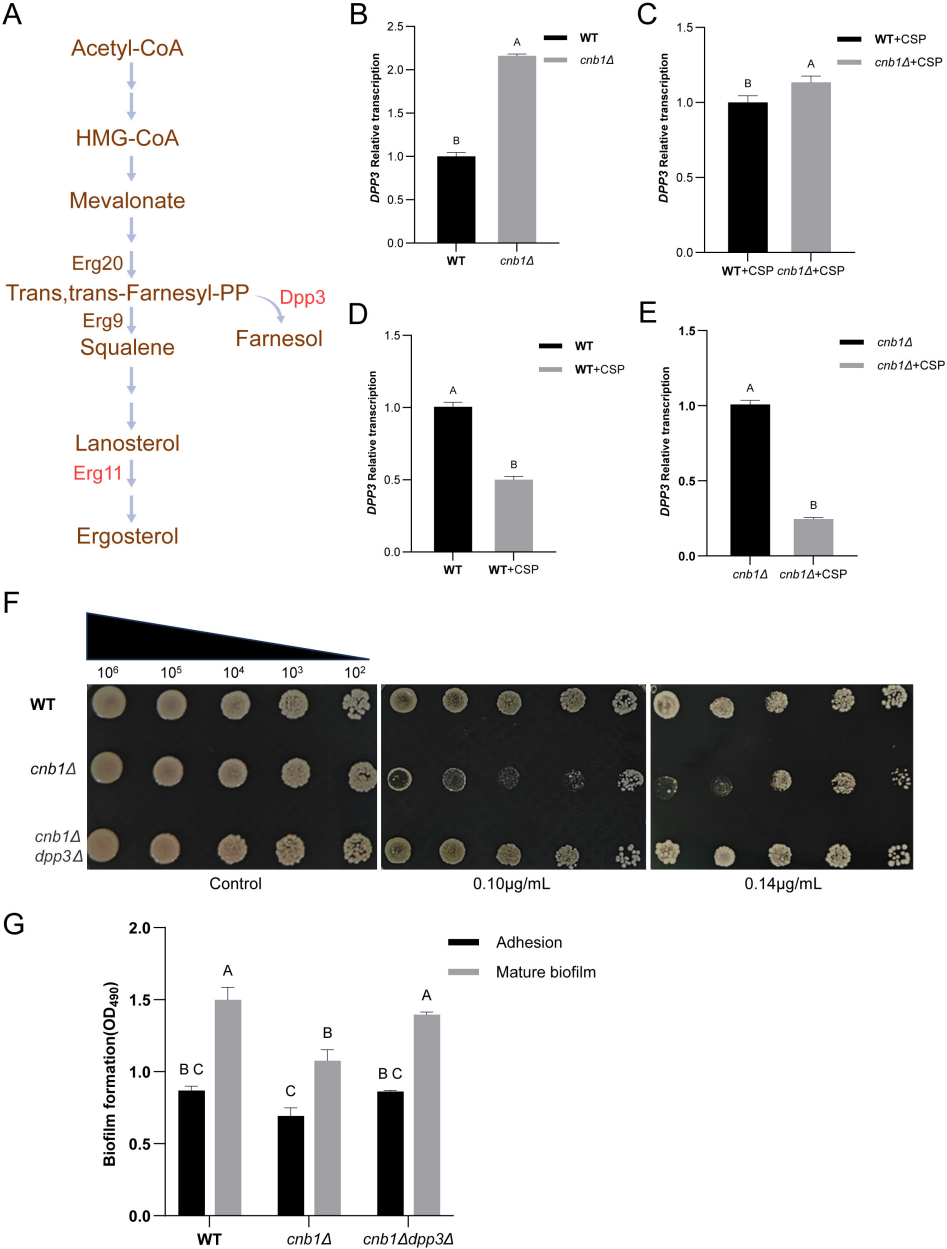
Involvement of Dpp3 in quorum sensing and biofilm formation in the calcineurin mutant. **(A)** Schematic representation of the ergosterol and farnesol metabolic pathways. (**B-C)** Comparison of transcriptional levels between the wildtype (WT) strain and the calcineurin mutant (*cnb1Δ*) under conditions (**B)** without or (**C)** with 0.01 µg/ml caspofungin. (**D-E)** Comparison of transcriptional levels between conditions without and with 0.01 µg/ml caspofungin in (**D)** the WT strain and (**E)** the calcineurin mutant. (**F)** Spot assay for the WT, calcineurin mutant (*cnb1Δ*), and *cnb1Δdpp3Δ* strains in the presence of 0.01 µg/ml or 0.014 µg/ml caspofungin. (**G)** Biofilm formation assay for the WT, calcineurin mutant (*cnb1Δ*), and *cnb1Δdpp3Δ* strains. Standard deviations were calculated from three independent replicates. Capital letters (**A, B, C**) above the data bars indicate statistical significance: bars sharing the same letter denote *P*>0.05, while bars with different letters denote *P*<0.05.

**Figure 4:**
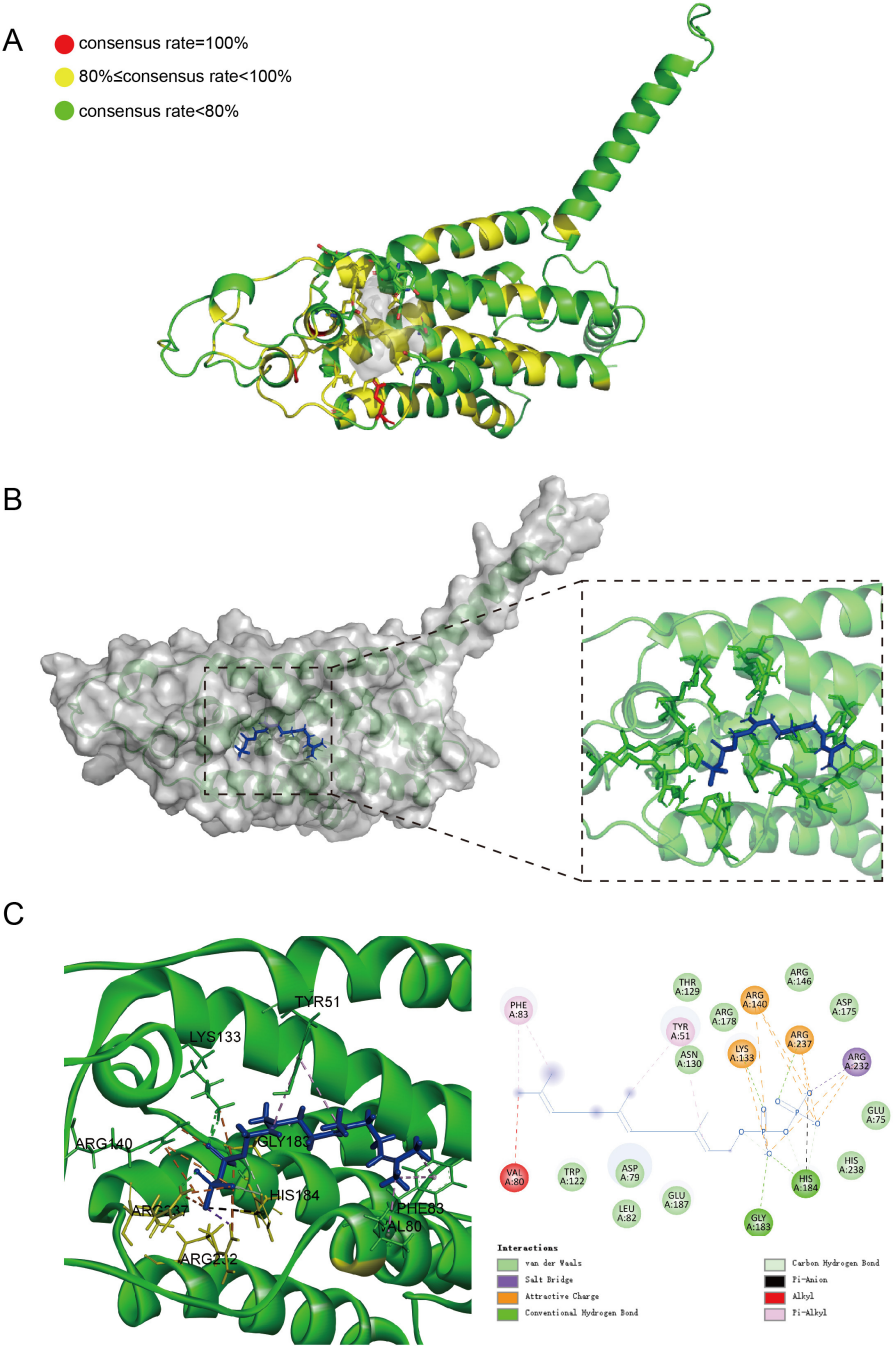
Structural and functional analysis of *N. glabrata* Dpp3. (**A**) Three-dimensional structural model of *N. glabrata* Dpp3 protein. The conserved sequences were visualized using PyMol, with yellow indicating highly conserved regions (≥80% similarity, see Supplementary Figure S3B) and red representing absolutely conserved amino acid residues (100% similarity, see Supplementary Figure S3B). The predicted catalytic pocket (shown in gray) was identified using the CASTpFold web server (https://cfold.bme.uic.edu/castpfold/) and rendered with PyMol. (**B**) Molecular docking model of *N. glabrata* Dpp3 protein with substrate FPP. The docking simulation was performed using YASARA software. (**C**) Interaction forces between the amino acid residues of Dpp3 and the substrate FPP, analyzed and visualized using Discovery Studio 2021 (BIOVIA).

Farnesol is biosynthesized as a metabolic shunt of ergosterol from farnesyl pyrophosphate (FPP) through dephosphorylation (Figure 3A). In *C. albicans*, only ∼1.6% of FPP is converted to farnesol, despite the significant QS effect it exerts [23]. The conversion of FPP to farnesol is catalyzed by the FPP phosphatase Dpp3 in *C. albicans* (CaDpp3). The putative *DPP3* of *N. glabrata* was first aligned with *CaDPP3* (Supplementary Figure S3A). The alignment of amino acid sequences demonstrated highly conserved secondary structures between the two Dpp3 proteins (Supplementary Figure S3B). The amino acid consensus was analyzed through cross-genus alignment (Supplementary Figure S4). A homology model of Dpp3 was generated using AlphaFold3, and its catalytic pocket was predicted using CASTpFold [24] (Supplementary Figure S5). Amino acid residues with a high consensus rate were predominantly localized surrounding the active domain or within conserved secondary structures (Figure 4A). The structural model was further validated through molecular docking of FPP into the predicted catalytic site of Dpp3 (Figure 4B). The phosphate groups of FPP exhibited strong and intricate interactions with residues in the active pocket (simulated by Discovery Studio, see Figure 4C), which may contribute to substrate recognition and the dephosphorylation of FPP to farnesol.

The qPCR (quantitative PCR) results showed that Dpp3 mRNA was more than twice as abundant in the calcineurin mutant compared with the WT strain (Figure 3B). The mRNA levels of *DPP3* remain significantly higher in the calcineurin mutant compared with the WT strain following the addition of caspofungin (Figure 3C). However, surprisingly, the addition of caspofungin significantly reduced the transcription of *DPP3* (Figure 3D and E). Paradoxically, caspofungin treatment induces a farnesol-dependent QS response in *cnb1Δ* cells. This may imply the involvement of tight regulatory mechanisms to maintain cellular homeostasis through the QS effect. On the other hand, the caspofungin-induced QS effect was most probably not directly induced by up-regulating Dpp3, which might not be the rate-limiting enzyme in synthesizing farnesol. We subsequently deleted *DPP3* in the calcineurin mutant. No farnesol can be detected in the *cnb1Δdpp3Δ* mutant, and the QS-like effect was completely abolished in both the spot assay (Figure 3F) and the biofilm assay (Figure 2F). In addition, the deletion of *DPP3* restored the diminished biofilm formation in the calcineurin mutant when caspofungin was not treated (Figure 3G). This suggests that beyond its role in the caspofungin-triggered QS response, Dpp3 may regulate biofilm formation via other metabolic pathways.

### *DPP3* is involved in ergosterol metabolism and membrane stress tolerance

**Figure 5:**
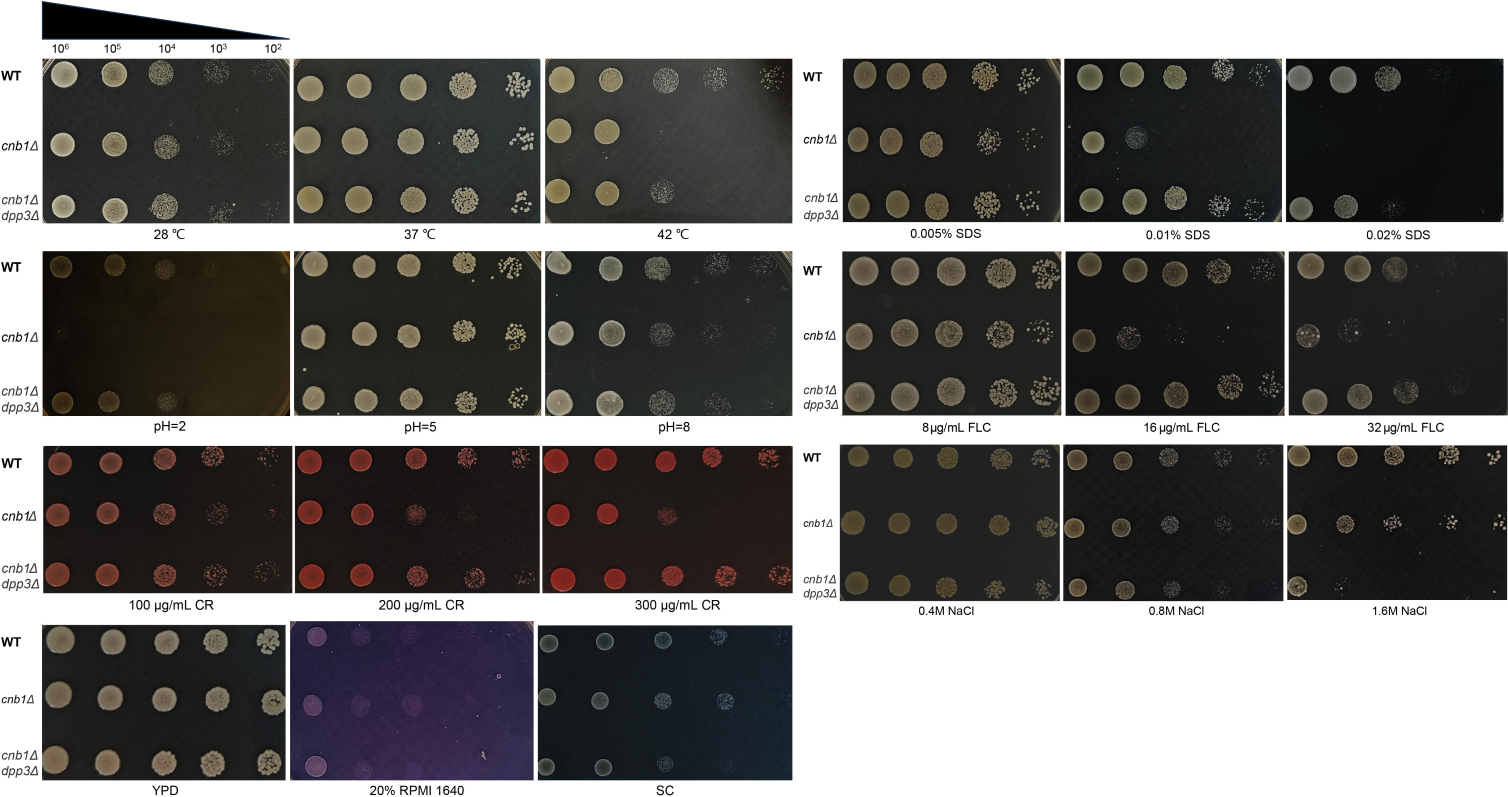
Spot assays to assess the growth of wildtype (WT), calcineurin mutant (*cnb1Δ*), and *cnb1Δdpp3Δ* strains under various conditions. Growth was evaluated under different temperatures: 28°C, 37°C, and 42°C; pH levels: 2, 5, and 8; SDS concentrations: 0.005%, 0.01%, and 0.02%; fluconazole (FLC) concentrations: 8 µg/ml, 16 µg/ml, and 32 µg/ml; Congo red (CR) concentrations: 100 µg/ml, 200 µg/ml, and 300 µg/ml; sodium chloride (NaCl) concentrations: 0.4 M, 0.8 M, and 1.6 M; media: YPD, 20% RPMI, and SC. The indicated conditions were used to compare the growth responses of the WT, *cnb1Δ*, and *cnb1Δdpp3Δ* strains. YPD, yeast extract peptone dextrose; SC, synthetic complete; FLC, fluconazole; CR, Congo red; NaCl, sodium chloride.

**Figure 6:**
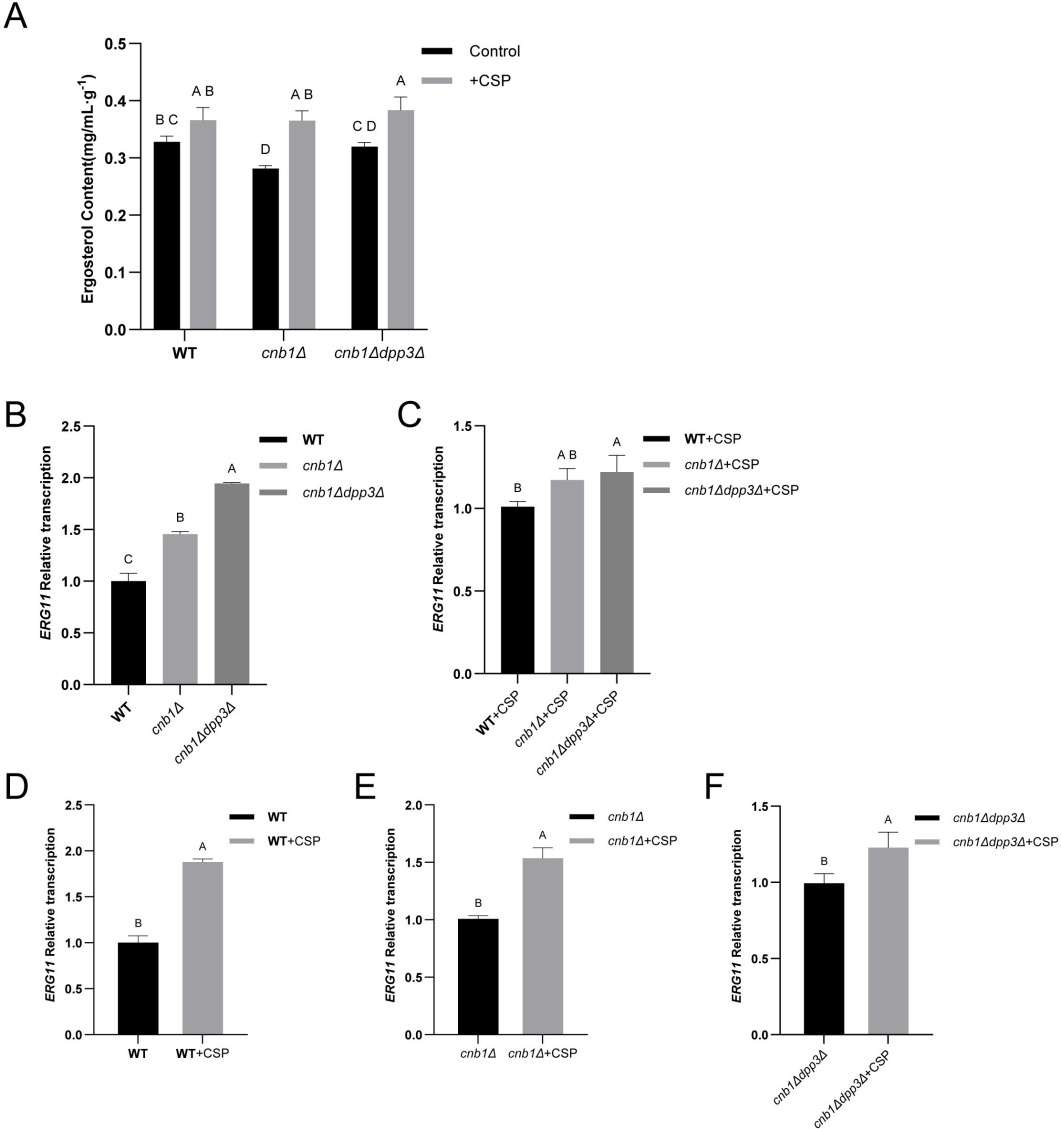
Influence of calcineurin and caspofungin on ergosterol accumulation and *ERG11* transcription. **(A)** Ergosterol accumulation in the wildtype (WT), *cnb1Δ*, and *cnb1Δdpp3Δ* strains with or without caspofungin (0.01 µg/ml) treatment. (**B-C)** qPCR analysis comparing *ERG11* transcription levels in the WT, *cnb1Δ*, and *cnb1Δdpp3Δ* strains (**B)** without or (**C)** with caspofungin (0.01 µg/ml) treatment. (**D-F)** Impact of caspofungin (0.01 µg/ml) on *ERG11* transcription in the (**D)** WT, (**E)** *cnb1Δ*, and (**F)** *cnb1Δdpp3Δ* strains. Standard deviations were calculated from three independent replicates. Capital letters (**A, B, C, D**) above the data bars indicate statistical significance: bars sharing the same letter denote *P*>0.05, while bars with different letters denote *P*<0.05.

**Figure 7:**
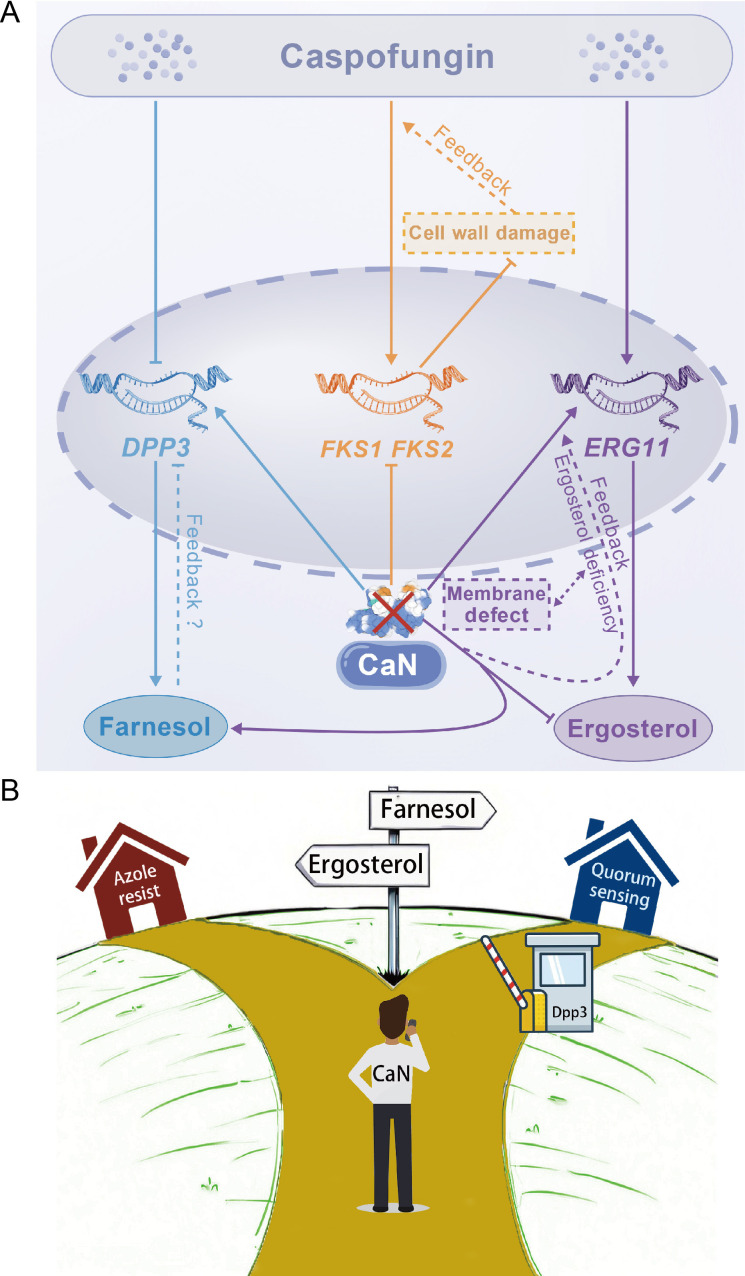
Schematic diagram illustrating the metabolic regulation of calcineurin and caspofungin to the farnesol–ergosterol pathway and β-glucan synthesis. **(A)** Schematic diagram illustrating the impact of calcineurin deletion and sub-MIC caspofungin treatment on the transcription of *DPP3* **,** *FKS1* **,** *FKS2* **,** and *ERG11*. Solid arrows represent regulatory relationships supported by qPCR results or the known roles of the corresponding enzymes in metabolic pathways. Dashed lines indicate proposed regulatory interactions based on experimental observations and hypothesized mechanisms. (**B)** Schematic diagram illustrating the role of calcineurin in fine-tuning the metabolic flux of ergosterol and farnesol, so as its consequential contributions to azole resistance and quorum sensing. CaN, calcineurin.

Phenotypically, it has been shown that farnesol reduces fluconazole resistance, as evidenced by the decreased fluconazole MIC (Supplementary Figure S1). The WT, calcineurin mutant, and *cnb1Δdpp3Δ* mutant strains were tested under various media and conditions. We observed that the deletion of *DPP3* restored the *cnb1Δ* mutant’s resistance to low pH, membrane stress (SDS and fluconazole), cell wall stress (Congo red), and high temperature (Figure 5). The restored resistance to fluconazole was consistent with the restored ergosterol accumulation (Figure 6A). However, surprisingly, the transcription of the fluconazole target Erg11 was significantly increased by the deletion of calcineurin and further increased by the additional deletion of *DPP3* (Figure 6B and C). The addition of caspofungin also significantly enhanced the transcription of Erg11 (Figure 6D, E and F). These results indicate a highly complex regulatory interaction between calcineurin, caspofungin, mevalonate–ergosterol pathway, and mevalonate–farnesol metabolism (Figure 7A). In the calcineurin mutant, reduced ergosterol accumulation and its consequential phenotype of increased sensitivity to fluconazole, along with elevated farnesol secretion, are all abolished by the deletion of the FPP dephosphorylase Dpp3. These findings collectively highlight the critical role of calcineurin in regulating this essential metabolic pathway (Figure 7B).

### The QS cells accompanied by compromised cell wall integrity

**Figure 8:**
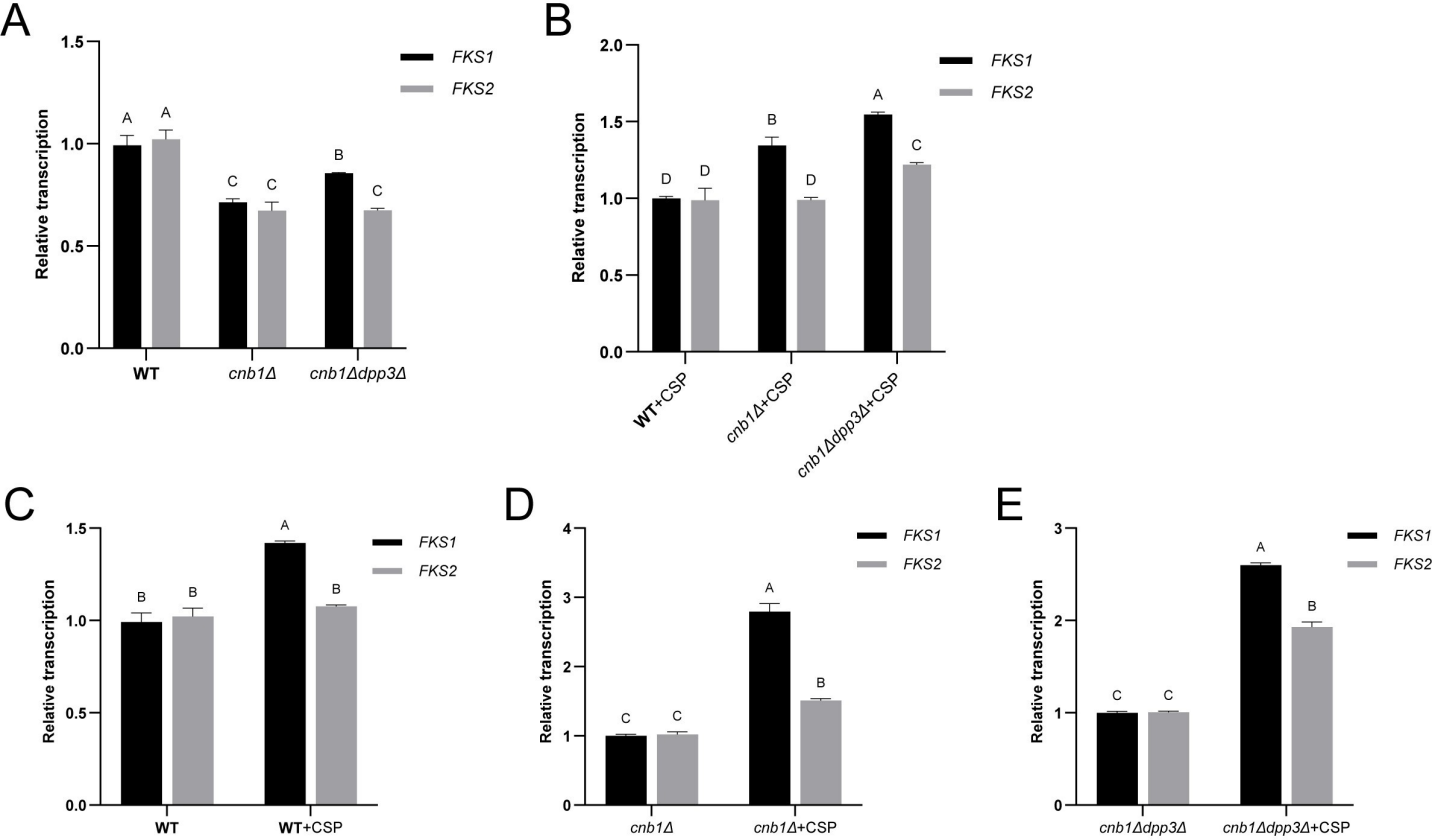
Influence of calcineurin and caspofungin on the transcription of *FKS* genes. **(A-B)** Transcription levels (qPCR) of *FKS1* and *FKS2* genes in the wildtype (WT), *cnb1Δ*, and *cnb1Δdpp3Δ* strains (**A)** without or (**B)** with 0.01 µg/ml caspofungin treatment. (**C-E)** Impact of caspofungin (0.01 µg/ml) on the transcription (by qPCR) of *FKS1* and *FKS2* genes in the (**C)** WT, (**D)** *cnb1Δ*, and (**E)** *cnb1Δdpp3Δ* strains. Standard deviations were calculated from three independent replicates. Capital letters (**A, B, C, D**) above the data bars indicate statistical significance: bars sharing the same letter denote *P*>0.05, while bars with different letters denote *P*<0.05.

Calcineurin-Crz1 mediates the expression of cell wall biosynthesis-related genes, including *FKS1*, *DCW1*, *FLC1* [25], and *FKS2* [26,27]. Meanwhile, farnesol influences the morphology and structure of the *C. albicans* cell wall by regulating glucan-related genes such as *PHR2*, *PIR1*, and *GSC1*[28]. The spot assay indicated that the calcineurin mutant was sensitive to cell wall stress, while further deletion of *DPP3* significantly restored its resistance. The expression of *FKS1* and *FKS2*, which encode the echinocandin target β-(1,3)-glucan synthase, was evaluated in WT, *cnb1Δ* mutants, and *cnb1Δdpp3Δ* double mutants upon caspofungin induction. Expression of both *FKS1* and *FKS2* was significantly down-regulated upon deletion of calcineurin, while additional deletion of *DPP3* partially restored *FKS1* transcription (Figure 8A). Upon caspofungin treatment, *FKS1* transcription was up-regulated in the WT strain. In contrast, both the *cnb1Δ* and *cnb1Δdpp3Δ* double mutants exhibited significant transcriptional induction of both *FKS1* and *FKS2*, with the effect being more pronounced (Figure 8C, D and E). Eventually, with the addition of caspofungin, the mRNA levels of *FKS1* in *cnb1Δ*, and of both *FKS1* and *FKS2* in *cnb1Δdpp3Δ* exceeded those of WT strain (Figure 8B). This suggests that the cells may have a mechanism to protectively up-regulate the expression of these genes when Fks proteins were targeted and the ‘sick’ cell wall was formed (Figure 7A).

**Figure 9:**
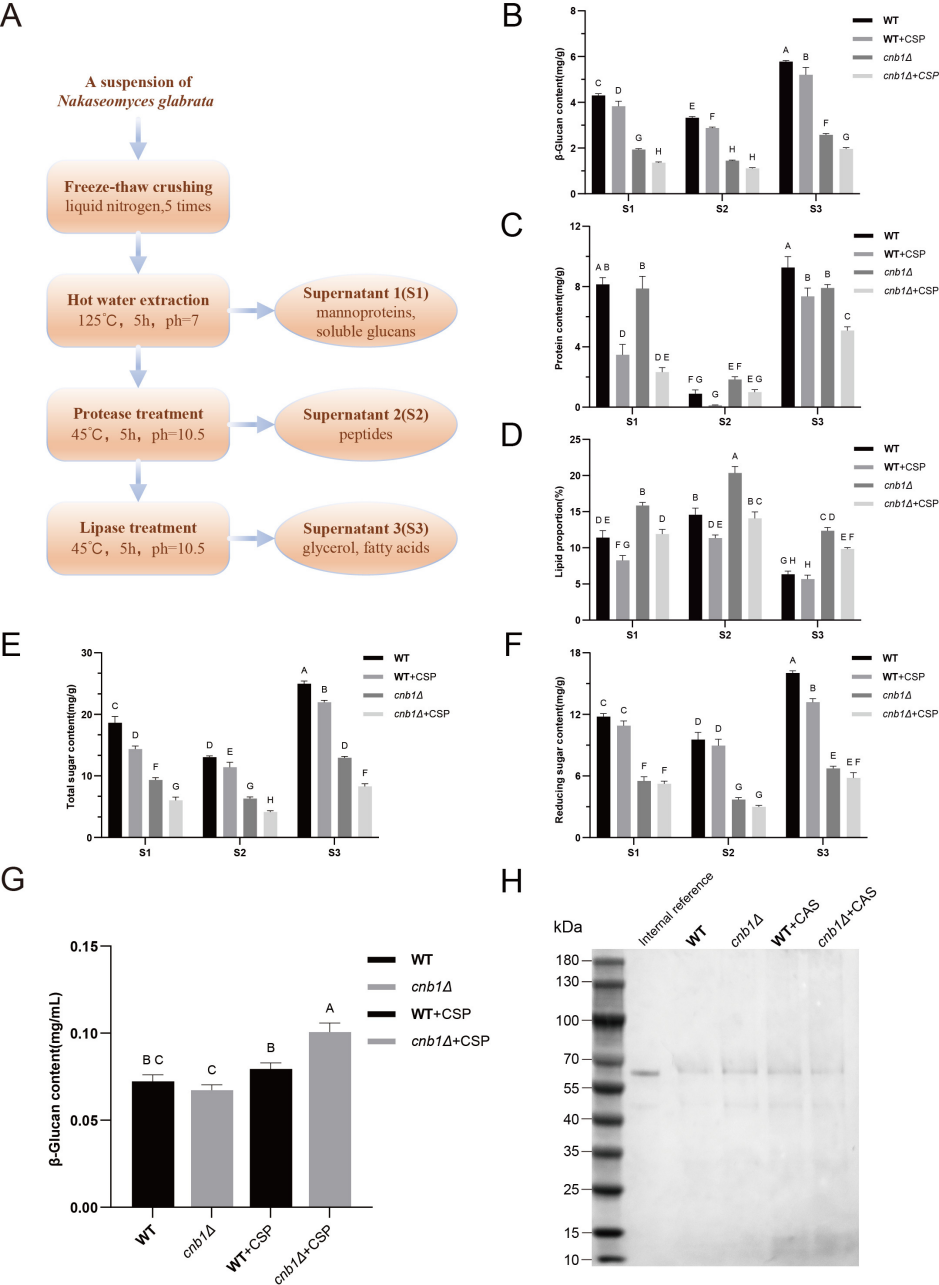
Effects of calcineurin deletion and caspofungin on cell wall compositions. **(A)** Schematic diagram of the cell wall separation process based on the protocol developed by Chema et al. (2014). (**B-F)** Content of (**B)** β-glucan, (**C)** protein, (**D)** lipid, (**E)** total sugar, and (**F)** reducing sugar in different cell wall fractions of the wildtype (WT) and *cnb1Δ* strains, with or without caspofungin (0.01 µg/ml) treatment. (**G)** β-glucan content in the fermentation medium of the WT and *cnb1Δ* strains, with or without caspofungin treatment. (**H)** SDS-PAGE followed by Coomassie staining of the concentrated medium from the WT and *cnb1Δ* strains, with or without caspofungin treatment. Loading dye (Beyotime®, P0015L) was used to facilitate sample loading and monitor the electrophoresis process, while T4 ligase (Beyotime®, D7008, sized between 55–70 kDa) was added at a concentration of 0.025 mg/ml to serve as an internal reference for normalizing sample dilution and loading. Internal reference: 0.025 mg/ml T4 ligase. Standard deviations were calculated from three independent replicates. Capital letters (**A, B, C, D, E, F, G, H**) above the data bars indicate statistical significance: bars sharing the same letter denote *P*>0.05, while bars with different letters denote *P*<0.05.

We also determined the β-glucan, protein, sugar, and lipid content in different cell wall fractions using a standardized protocol [29] (Figure 9A). The results revealed that the β-glucan content was significantly lower in the calcineurin mutant compared with the WT strain, and this component was notably reduced in both strains upon caspofungin treatment (Figure 9B). The protein content in the cell wall was significantly reduced following caspofungin treatment (Figure 9C). Surprisingly, however, the lipid content in the cell wall of *cnb1Δ* was significantly increased, while it decreased significantly upon addition of caspofungin (Figure 9D). In general, the *cnb1Δ* exhibited significantly lower levels of both total sugar (Figure 9E) and reducing sugar (Figure 9F) compared with the WT strain, while caspofungin-treated cells showed a reduction in total sugar content. These findings suggest that the cell wall compositions undergo substantial changes upon deletion of calcineurin and/or addition of caspofungin. In addition to the notable alterations in cell wall compositions, we also investigated specific components potentially released from the cell wall into the extracellular medium. Following caspofungin treatment, the calcineurin mutant exhibited significantly elevated levels of β-glucan, a major cell wall component, in the extracellular medium (Figure 9G). Additionally, caspofungin treatment led to a substantial increase in short peptides released into the medium (Figure 9H).

## Discussion

QS is a density-dependent communication mechanism in which cells secrete QSMs, some of which inhibit cell growth. Notably, studies have shown that in fungi, the QSM farnesol can suppress biofilm formation [30,31]. However, the conditions required to induce QS effects, beyond high cell density, remain poorly understood, particularly in terms of their molecular basis. For instance, it remains unclear whether antifungals can trigger QS or how these effects are intrinsically linked to metabolic regulation. On the other hand, calcineurin has been extensively studied and is considered a highly potential antifungal target, particularly in combination with other antifungals [32]. These studies mainly rely on phenotypic assays and have never been directly associated with QS effects.

**Figure 10:**
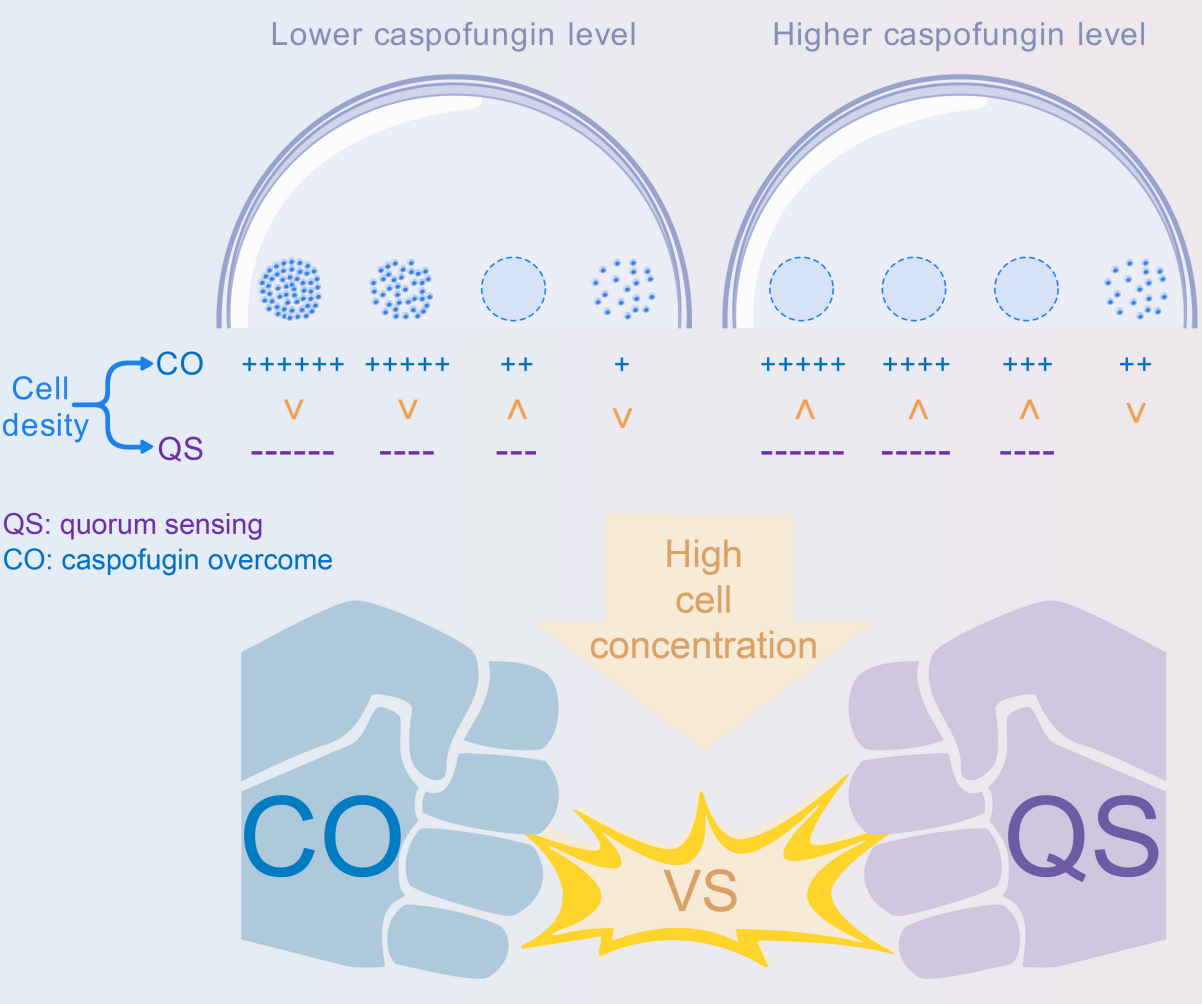
Schematic diagram illustrating the antagonistic effect between high cell density-induced quorum sensing and caspofungin resistance.

We demonstrate that targeting calcineurin may synergize with caspofungin to induce a QS effect mediated by farnesol. This QS effect depends on calcineurin deficiency, sub-MIC levels of caspofungin, and a high-density cell population. High cell density can elicit two antagonistic effects: overcoming antibiotic inhibition and QS-mediated growth inhibition, which positively and negatively regulate cell propagation (Figure 10). These results were consistently observed in both spot assays and biofilm formation assays. The QS-mediated growth inhibition exhibited a striking dependence on a narrow caspofungin concentration window. This sensitivity reflects the delicate balance between the drug’s sub-inhibitory induction of QS and its outright fungicidal activity. At a threshold concentration of 0.1–0.14 µg/ml (Figure 2A), the signaling threshold for a communal growth arrest is met or exceeded across higher cell densities. This behavior underscores how QS can function as a deterministic switch between distinct physiological states in response to minimal environmental changes, a phenomenon that would be masked in a conventional MIC assay. Farnesol was detectable only in the calcineurin mutant, where the transcription of the farnesol synthase gene *DPP3* was significantly up-regulated, and deletion of *DPP3* abolished the QS effect. Deletion of *DPP3* restored the calcineurin mutant’s biofilm formation to a WT-like manner, likely because the absence of farnesol eliminated its antibiofilm effect, as previously reported [33]. The QS effect observed in the calcineurin mutant was replicated by treating cells with FK506, a calcineurin inhibitor, suggesting that calcineurin represents a promising antifungal target, particularly in cases where caspofungin fails to kill cells due to intrinsic or acquired resistance (in our study, at sub-MIC levels). However, this mechanism might work in a very tricky way, as it operates in a cell density-dependent manner. A potential application is in anti-biofilm strategies, especially since biofilms are the dominant form of troublesome pathogenic microbes in our bodies or on clinical devices. Considering the puzzling ‘paradoxical growth’ of caspofungin and the cell density-dependent resistance to antibiotics, the development of a smart and effective drug treatment strategy becomes even more challenging [22].

Calcineurin regulates a wide range of biological processes, including various stress responses, which are conventionally thought to be mediated through Crz1 [34]. We found that deletion of *DPP3* significantly restored the calcineurin mutant’s resistance to cell wall stress, cell membrane stress, high temperature, and low pH. Additionally, deletion of *DPP3* rescued ergosterol accumulation in the calcineurin mutant, thereby restoring its resistance to fluconazole. This is attributed to the fact that farnesol and ergosterol are synthesized as metabolic shunts. The recovery of the calcineurin mutant against multi-stresses, mediated by *DPP3* deletion, should be independent of the transcription activator Crz1, the hallmark downstream target of calcineurin. This is evidenced by the fact that, as previously reported, the deletion of *CRZ1* does not reduce azole resistance or ergosterol accumulation, indicating that Crz1 likely regulates this metabolic pathway in an opposite manner than calcineurin. Therefore, this study also reveals a calcineurin-regulated, ergosterol- and farnesol-dependent, yet Crz1-independent, multi-stress resistance mechanism. Surprisingly, although the calcineurin mutant accumulated less ergosterol, the transcription of Erg11, a key ergosterol synthase and the target of azoles, was increased in the calcineurin mutant and was even higher in the *cnb1Δdpp3Δ* strain. Furthermore, caspofungin inhibited the transcription of *DPP3* while inducing *ERG11* transcription. These findings suggested the existence of a complex feedback system regulating the interplay between ergosterol and farnesol metabolism in response to QS and membrane defects.

**Figure 11:**
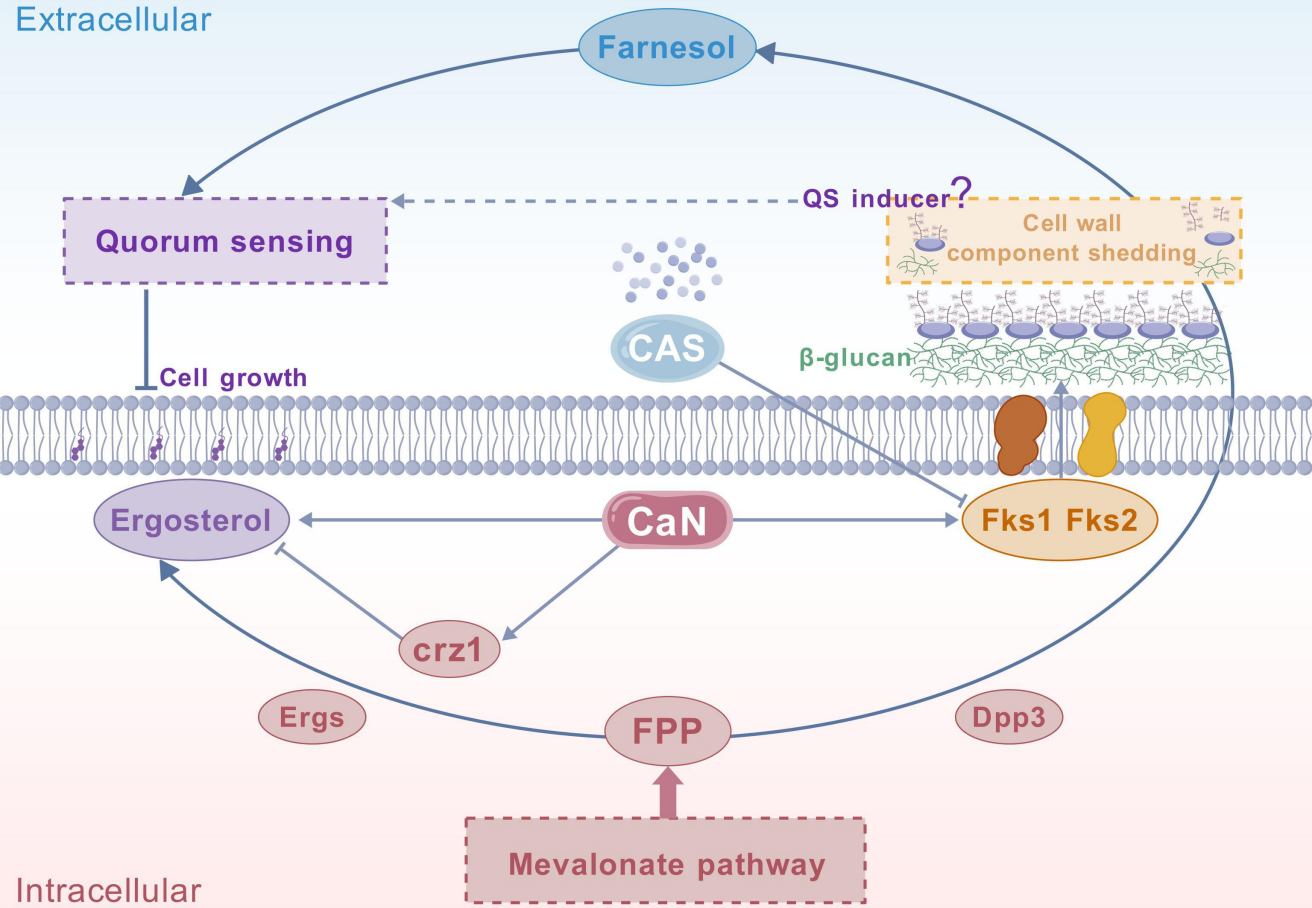
Schematic diagram of intracellular metabolic regulation and its proposed influence on extracellular quorum sensing (QS) induction.

Deletion of *DPP3* also restored the transcription of *FKS* genes in the calcineurin mutant, explaining its recovery of resistance to cell wall stress. The addition of caspofungin further induced the transcription of *FKS* genes, suggesting a feedback mechanism in response to Fks targeting. QS can be triggered by high cell density, leading to the release of QSMs to the medium. When QSMs reach a threshold concentration, they can induce the QS effect. In the context of the hypersensitivity of the calcineurin mutant to cell wall stress, and considering that caspofungin is a cell wall-targeting antifungal, we preliminarily hypothesize that a compromised cell wall structure may release compounds that could initiate the QS effect. In fact, higher cell density likely results in increased cell debris, which could possibly contain these QS-inducing compounds. As an example, the leucine-rich repeat protein domain has been implicated in sensing cell wall fragments in *Arabidopsis* and *C. albicans* [35,36]*.* Our results showed significant alterations in β-glucan, protein, lipid, and sugar compositions within the cell wall upon deletion of calcineurin or addition of caspofungin, while the addition of caspofungin increased β-glucan and short peptides in the medium. Figure 11 schematically represents the proposed intracellular metabolic regulations and QS induction.

The schematic diagram is drawn by BioGDP [37]. CaN, calcineurin.

## Materials and methods

### Yeast strains, media, and culture conditions

The WT strain *N. glabrata* ATCC2001 and the calcineurin mutant (*cnb1Δ*) were obtained from Miyazaki et al. [38]. *N. glabrata* was grown in yeast extract peptone dextrose medium (YPD), synthetic complete medium (SC), or RPMI 1640 medium. The liquid cultures were grown at 37°C with shaking at 180 rpm. Each strain is activated overnight and normalized to an optical density of OD_600_=0.05 for inoculation. Caspofungin and fluconazole used in this work were purchased from Macklin^®^, Shanghai, and Adamas Life^®^, Shanghai.

### Spot test

Cells were harvested from YPD culture at the logarithmic phase and diluted to an OD_600_ of 1, followed by ten-fold serial dilutions. The diluted cell suspension was spotted on a YPD plate containing 0.01 or 0.014 µg/ml caspofungin (or other stress conditions) and grew at 37°C for 24–48 h [39]. To avoid the confounding effects of caspofungin’s intrinsic antifungal activity, both the WT and mutant strains were treated with a sub-MIC concentration of the drug (See Supplementary Figure S1, 0.3 μg/ml MIC for the calcineurin mutant). This approach allowed us to isolate and demonstrate that the observed growth inhibition was attributable to QS (or the high inoculum concentration) rather than the drug itself. Stress tolerance was tested using spot assays under various growth conditions, including different media (YPD, SC, and RPMI 1640) and different types of stress, including varying temperatures (28°C, 37°C, and 42°C), pH values (pH 3, 5, or 8), concentrations of sodium chloride (0.4M, 0.8M, and 1.6M), concentrations of SDS (0.005%, 0.01%, 0.02%, and 0.04%), and Congo Red (100 μg/ml, 200 μg/ml, and 300 μg/ml). These conditions were designed to assess whether a growth difference exists between the WT and mutant strains or whether both exhibit equally stressed growth under the challenge compared with the unstressed control.

### QS induction in liquid medium by FK506 and caspofungin

An overnight culture of the WT *N. glabrata* cells was used for a two-fold serial dilution in fresh YPD medium (final OD_600_=2, 1, 0.5, 0.25, 0.125, 0.0625, 0.03125, 0.015625, and 0.0078 in a 200 µl total volume). Caspofungin was added to a final concentration of 0.14 µg/ml, while FK506 was added to a final concentration of 1 mg/ml. The prepared cultures were thoroughly mixed in a 96-well plate and incubated at 37°C for 24 h. Following incubation, the cultures were resuspended by pipetting and subsequently diluted ten-fold (1:10) for OD_600_ measurement. The average optical density and standard deviations were calculated from three independent experiments.

### Extraction and detection of farnesol

Cells were inoculated from fresh overnight cultures, and the optical density was measured and adjusted to a starting OD_600_ of 0.05 by dilution with fresh medium. Cells were grown until reaching the logarithmic phase (14 h), after which caspofungin was added at a sub-MIC concentration of 0.1 μg/ml, followed by continued incubation for 6–8 h. Dodecane (10% of the total volume) was added and mixed thoroughly for extraction. Withdraw 400 µl of the dodecane layer and centrifuge to collect the upper dodecane layer. The farnesol content was determined using GC-MS. Chromatographic (Thermo Scientific™ TRACE™ 1300) conditions: chromatographic separation is carried out on an HP-5 (Agilent J&W HP-5MS 12 m, 0.20 mm, 0.33 µm) column. The chromatographic conditions are as follows: 1 µl sample volume (no split flow) is used, the initial temperature is 100°C, the temperature is raised to the final temperature of 280°C at the speed of 15°C/min, and it is kept at this temperature for 5 min. Mass spectrometry (Thermo Scientific™ ISQ™ 7000) conditions: using electron bombardment ion (EI) as ion source, heating to 230℃, solvent delay of 5 min, scanning range of 40~400 m/z, using atlas to retrieve qualitative retention time, and selecting ions 69 and 81 for quantification.

### *In vitro* biofilm formation (XTT method)

Colonies of *N. glabrata* were picked from YPD plates and cultured in YPD medium overnight. After centrifugation, the cells were washed with sterile PBS and resuspended in RPMI 1640 medium to 1.0 × 10^6^ cells/ml (calculated based on OD_600_ value). An inoculum volume of 200 µl was added to a 96-well microplate and incubated at 37°C for 90 min to allow adhesion. The supernatant was discarded, and the wells were washed three times with PBS to remove non-adherent cells. Add 200 μl of fresh RPMI 1640 medium and continue culturing for 24 h to form mature biofilm. The supernatant was discarded, and the wells were washed three times with PBS. One hundred microliters of XTT/menadione solution [2,3-bis-(2-methoxy-4-nitro-5-sulfophenyl)-2H-tetrazolium-5-carboxanilide: Yuan-Ye^®^, Shanghai] was added, and the plate was incubated at 37°C in the dark for 2 h. After incubation, 75 µl of supernatant from each well was transferred to a new microtiter plate, and the OD_490_ was measured using a microplate reader (Agilent^TM^ BioTek Synergy H1). The metabolic activity of the biofilm after adhesion was directly measured by the XTT method after 90 min of incubation without further incubation for 24 h.

### Inoculum-gradient and caspofungin-gradient biofilm assay

Biofilm formation experiments were conducted using a range of inoculum concentrations, from low to high (OD_600_=0.0625, 0.125, 0.25, 0.5, 0.75, 1, 1.5, 2). Caspofungin was added after the adhesion period (90 min) in fresh YPD medium, using two-fold serial dilutions to achieve final concentrations ranging from 0.009375 µg/ml to 4.8 µg/ml. The subsequent experimental steps were consistent with those described for the biofilm-XTT assay.

### Construction of the *DPP3Δ* strain

Gene knockout was performed using homologous recombination, where the *DPP3* gene (systemic name: GVI51_H00957) was replaced by the hygromycin B resistance cassette. The 5′ flanking region of *DPP3* was amplified from genomic DNA using primers G-a-F and G-a-R, while the 3′ flanking region was amplified using primers G-b-F and G-b-R. The resulting 5′ and 3′ flanking regions were subsequently cloned into the plasmid pTHT, flanking the hygromycin resistance marker DNA on either side, through One Step Cloning (ClonExpress Ultra One Step Cloning Kit, Vazyme). The hygromycin B resistance cassette was subsequently amplified from the constructed plasmid using primers G-a-F and G-b-R. Electroporation (0.2 cm cuvette, 1.5 kV) was performed to transform the cassette into competent cells [40]. The transformation was plated on hygromycin B supplemented YPD plates for selection. Colonies were verified using PCR and sequencing. The primers used have been listed in the Supplementary Table S1.

### Determination of ergosterol

Cells were grown to the logarithmic phase (14 h) in shaking flasks. Caspofungin (sub-MIC) was added, and the cultures were incubated for 6–8 h. After centrifugation at 8,000 rpm for 10 min, the cells were collected. Then, 20 ml of an ethanol-50% potassium hydroxide solution (3:2) was added and subsequently heated at 85°C for 3 h for extraction. After cooling to room temperature, 15 ml of n-hexane was added, and the solution was shaken vigorously for 30 min and allowed to stand for phase separation. The upper phase (7.5 ml) was concentrated under speed-vacuum, and 2 ml of ethanol was used to dissolve the samples [41]. The ergosterol content was determined by high-performance liquid chromatography (Thermo Scientific™ UltiMate™ 3000) with a C18 column (J&K Scientific™ C18 WR, 150 × 4.6 mm, 5 µm) at 28°C. The mobile phase consisted of 97% methanol, and the UV detection wavelength was set to 283 nm with a retention time of 15 min.

### Determination of cell wall components

Cells were inoculated in YPD medium and cultured to the logarithmic growth phase (14 h). Caspofungin was added at a sub-MIC concentration of 0.1 μg/ml. This culture was incubated for another 6–8 h. Cells were collected by centrifugation and freeze-thaw-wash five times. The insoluble residue was freeze-dried and weighed to obtain the precipitate. The extraction and components determination methods were adapted according to the protocol established by Chema et al. [29]. The precipitate was suspended with ultrapure water to a 6.7% (w/v) solution and adjusted the pH to 7 using 9.98 M sodium hydroxide. The suspension was heated to 125°C for 5 h. After cooling down, it was centrifuged to collect the supernatant and then washed twice with ultrapure water (5 ml each time), and all the supernatant was collected to obtain S1. The precipitate was freeze-dried and weighed and subsequently diluted with distilled water to 6.7% (w/v) and gently stirred, heated to 45°C, and adjusted the pH to 10.5 using 9.98 M sodium hydroxide. Proteinase (Macklin^®^, Shanghai) was added at 0.17% (v/v) at 0, 1.5, and 3 h. After a total of 5 h incubation, the supernatant was collected by centrifugation, followed by twice water wash (5 ml), and all the supernatant was collected to obtain S2. The precipitate was freeze-dried and weighed again and subsequently diluted with ultrapure water to 4% (w/v) and gently stirred at 45°C, pH 10.5. Lipase (Macklin^®^, Shanghai) was added at 0.10% (v/v) and incubated for 3 h. After centrifugation, the supernatant was collected and water washed (5 ml) twice. All the supernatant was collected to obtain S3.

The β-glucan content was determined using the β-Glucan Assay Kit (Megazyme International Ireland Ltd., Wicklow, Ireland). The protein content was determined using the Bicinchoninic Acid Assay Kit (BCA, Beyotime Biotechnology Shanghai Ltd., Shanghai, China). For lipid determination, 0.1 g of the liquid sample was weighed and added with 0.1 ml of hydrogen chloride. This sample was incubated in a 70–80°C water bath for 40–50 min for full digestion. Then, 0.1 ml of ethanol was added and mixed thoroughly. After cooling down, the mixture was washed several times with a total of 0.25 ml of ether. All the liquid was collected. After 1 min of shaking, the lid was opened to evaporate and closed to let it stand for 12 min. The prepared sample was then washed with a mixture of petroleum ether and ether (1:1) and sand for phase separation. The upper liquid was removed, followed by adding 0.05 ml of ether, shaking, and standing to remove the ether layer. The remaining liquid was collected, evaporated, and oven-dried for 2 h. After cooling down in a desiccator for 30 min, the leftover was weighed to obtain the lipid weight.

The 3,5-dinitrosalicylic acid (DNS) reagent colorimetric method is used for the determination of sugar content. To determine the reducing sugar content, 200 μl of the liquid sample was adjusted to pH 7.4, mixed with 400 μl of DNS reagent (Codow^®^, Guangzhou), and heated in a boiling water bath for 5 min. After cooling down, absorbance was measured at 540 nm, and reducing sugar concentration was calculated using a glucose standard curve. For total sugar analysis, 200 μl of sample was first acid-hydrolyzed by adding 100 μl of 6M hydrogen chloride and heating at 100°C for 30 min with occasional stirring. The hydrolyzed sample was neutralized to pH 7.4 with 6M sodium hydroxide diluted to 2 ml with ultrapure water for reducing sugar determination.

### Verification of medium protein and β-glucan

Cells were grown in SC medium and grew to the logarithmic phase (14 h). Caspofungin was added to a sub-MIC concentration of 0.1 µg/ml. The cultures were incubated for another 6–8 h. After thorough centrifugation, 10 ml of supernatant was collected and lyophilized and subsequently dissolved in 200 µl of ultrapure water. The samples were separated using SDS-PAGE. To quantify the medium β-glucan content, 900 µl of acetate buffer (pH 4.8) was used to dissolve the lyophilized sample, and glucose concentration was measured using a glucose detection kit (O-toluidine method). The β-glucan was digested using 100 µl of 10% β-glucanase and β-glucosidase mixture (1:10, Yuan-Ye^®^, Shanghai) and incubated at 50°C for 30 min. Glucose concentrations were measured in the samples both before and after enzymatic hydrolysis to eliminate the background, and the β-glucan concentration was calculated using a dehydration conversion factor of 0.9.

### Real-time PCR analysis

The exponentially growing cells were treated with or without 0.1 µg/ml caspofungin for 6 h. RNA was extracted using the FastPure^®^ Cell/Tissue Total RNA Isolation Kit, and cDNA was synthesized using HiScript^®^ II Q RT SuperMix for qPCR (+gDNA wiper). Real-time PCR was performed using the StepOnePlus™ Real-Time PCR System and ChamQ Universal SYBR qPCR Master Mix. *ACT1* was used as a housekeeping gene, and the relative expression levels were calculated using the ΔΔCT method. Supplementary Table S1 lists the primers used for the qPCR.

### MICs using the broth dilution assay

The MIC was determined using the broth dilution method according to Clinical and Laboratory Standards Institute (CLSI) guideline M27-A4 [37]. Three colonies larger than 1 mm in diameter were picked from freshly prepared YPD plates, suspended in sterile water, and adjusted to a concentration of 5 × 10^6^ cells/ml, approximately equivalent to a 0.5 McFarland standard. The prepared inoculum was diluted 1,000-fold in YPD medium. A microtiter plate was used with the first column as a blank control, columns 2–11 as experimental wells, and the drugs were serially diluted in twofold concentrations. Column 12 contained sterile water as a positive control. The prepared inoculum was added to wells 2–12 (80 μl per well), and the experiment was repeated three times. The plate was incubated at 37°C for 36–48 h before observing the results.

## Supplementary material

online supplementary material 1.

## Data Availability

All data are contained within the manuscript. All data supporting the findings of this study are available from the corresponding author upon request.

## Open Access

This article has been published open access under our Subscribe to Open programme, made possible through the support of our subscribing institutions, learn more here: https://portlandpress.com/pages/open_access_options_and_prices#conditional

## Abbreviations

DNS: 3,5-dinitrosalicylic acid
FPP: farnesyl pyrophosphate
GC-MS: gas chromatography-mass spectrometry
MIC: minimum inhibitory concentration
QS: quorum sensing
QSMs: quorum sensing molecules
SC: synthetic complete
SDS: sodium dodecyl sulfate
WT: wildtype
YPD: yeast extract peptone dextrose

## References

[1] Askari F. Kaur R 2025 Candida glabrata: a tale of stealth and endurance ACS Infect. Dis. 11 4 20 10.1021/acsinfecdis.4c00477 39668745

[2] Lass-Flörl C. Kanj S.S. Govender N.P. Thompson G.R. 3rd Ostrosky-Zeichner L. Govrins M.A 2024 Invasive candidiasis Nat. Rev. Dis. Primers 10 20 10.1038/s41572-024-00503-3 38514673

[3] Carolus H. Sofras D. Boccarella G. Jacobs S. Biriukov V. Goossens L. et al. 2024 Collateral sensitivity counteracts the evolution of antifungal drug resistance in Candida auris Nat. Microbiol. 9 2954 2969 10.1038/s41564-024-01811-w 39472696 PMC7618254

[4] Pfaller M.A. Diekema D.J. Turnidge J.D. Castanheira M. Jones R.N 2019 Twenty years of the SENTRY antifungal surveillance program: results for *Candida* species from 1997-2016 Open Forum Infect. Dis. 6 S79 S94 10.1093/ofid/ofy358 30895218 PMC6419901

[5] Uppuluri P. Nett J. Heitman J. Andes D 2008 Synergistic effect of calcineurin inhibitors and fluconazole against Candida albicans biofilms Antimicrob. Agents Chemother. 52 1127 1132 10.1128/AAC.01397-07 18180354 PMC2258509

[6] Chen Y.-L. Konieczka J.H. Springer D.J. Bowen S.E. Zhang J. Silao F.G.S. et al. 2012 Convergent evolution of calcineurin pathway roles in thermotolerance and virulence in *Candida glabrata* G3 (Bethesda, Md.) 2 675 691 10.1534/g3.112.002279 22690377 PMC3362297

[7] Li W. Shrivastava M. Lu H. Jiang Y 2021 Calcium-calcineurin signaling pathway in Candida albicans: a potential drug target Microbiol. Res. 249 126786 10.1016/j.micres.2021.126786 33989979

[8] Cai L. Dalal C.K. Elowitz M.B 2008 Frequency-modulated nuclear localization bursts coordinate gene regulation Nature New Biol. 455 485 490 10.1038/nature07292 18818649 PMC2695983

[9] Cen Y. Dijck P 2016 Characterization of the Gpr1 and calcineurin dependent regulation of stress resistance in Candida glabrata and development of molecular tools in this organism In Ontwikkeling van Nieuwe Moleculaire Technieken En Onderzoek Naar de Gpr1 En Calcineurine-Afhankelijke Regulatie van Stressresistentie in Candida Glabrata

[10] Ene I.V. Walker L.A. Schiavone M. Lee K.K. Martin-Yken H. Dague E. et al. 2015 Cell wall remodeling enzymes modulate fungal cell wall elasticity and osmotic stress resistance MBio 6 e00986-15 10.1128/mBio.00986-15 26220968 PMC4551979

[11] Jiang L. Xu H. Gu Y. Wei L 2023 A glycosylated Phr1 protein is induced by calcium stress and its expression is positively controlled by the calcium/calcineurin signaling transcription factor Crz1 in Candida albicans Cell Commun. Signal 21 237 10.1186/s12964-023-01224-y 37723578 PMC10506259

[12] De Cesare G.B. Hafez A. Stead D. Llorens C. Munro C.A 2022 Biomarkers of caspofungin resistance in *Candida albicans* isolates: A proteomic approach Virulence 13 1005 1018 10.1080/21505594.2022.2081291 35730400 PMC9225221

[13] Cornely O.A. Bassetti M. Calandra T. Garbino J. Kullberg B.J. Lortholary O. et al. 2012 ESCMID* guideline for the diagnosis and management of Candida diseases 2012: non-neutropenic adult patients Clin. Microbiol. Infect. 18 Suppl 7 19 37 10.1111/1469-0691.12039 23137135

[14] Liu N.-N. Acosta-Zaldívar M. Qi W. Diray-Arce J. Walker L.A. Kottom T.J. et al. 2020 Phosphoric metabolites link phosphate import and polysaccharide biosynthesis for Candida albicans cell wall maintenance MBio 11 e03225-19 10.1128/mBio.03225-19 32184254 PMC7078483

[15] Eickhoff M.J. Bassler B.L 2018 SnapShot: bacterial quorum sensing Cell 174 1328 1328 10.1016/j.cell.2018.08.003 30142348

[16] Fuqua W.C. Winans S.C. Greenberg E.P 1994 Quorum sensing in bacteria: the LuxR-LuxI family of cell density-responsive transcriptional regulators J. Bacteriol. 176 269 275 10.1128/jb.176.2.269-275.1994 8288518 PMC205046

[17] Williams P. Winzer K. Chan W.C. Cámara M 2007 Look who’s talking: communication and quorum sensing in the bacterial world Philos. Trans. R. Soc. Lond., B, Biol. Sci. 362 1119 1134 10.1098/rstb.2007.2039 17360280 PMC2435577

[18] Hornby J.M. Jensen E.C. Lisec A.D. Tasto J.J. Jahnke B. Shoemaker R. et al. 2001 Quorum sensing in the dimorphic fungus Candida albicans is mediated by farnesol Appl. Environ. Microbiol. 67 2982 2992 10.1128/AEM.67.7.2982-2992.2001 11425711 PMC92970

[19] Cao Y.-Y. Cao Y.-B. Xu Z. Ying K. Li Y. Xie Y. et al. 2005 cDNA microarray analysis of differential gene expression in Candida albicans biofilm exposed to farnesol Antimicrob. Agents Chemother. 49 584 589 10.1128/AAC.49.2.584-589.2005 15673737 PMC547270

[20] Shirtliff M.E. Krom B.P. Meijering R.A.M. Peters B.M. Zhu J. Scheper M.A. et al. 2009 Farnesol-induced apoptosis in Candida albicans Antimicrob. Agents Chemother. 53 2392 2401 10.1128/AAC.01551-08 19364863 PMC2687256

[21] Jiang S. Li H. Zhang L. Mu W. Zhang Y. Chen T. et al. 2025 Generic Diagramming Platform (GDP): a comprehensive database of high-quality biomedical graphics Nucleic Acids Res. 53 D1670 D1676 10.1093/nar/gkae973 39470721 PMC11701665

[22] Prasetyoputri A. Jarrad A.M. Cooper M.A. Blaskovich M.A.T 2019 The eagle effect and antibiotic-induced persistence: two sides of the same coin? Trends Microbiol. 27 339 354 10.1016/j.tim.2018.10.007 30448198

[23] Mehmood A. Liu G. Wang X. Meng G. Wang C. Liu Y 2019 Fungal quorum-sensing molecules and inhibitors with potential antifungal activity: a review Molecules 24 1950 10.3390/molecules24101950 31117232 PMC6571750

[24] Ye B. Tian W. Wang B. Liang J 2024 CASTpFold: computed atlas of surface topography of the universe of protein folds Nucleic Acids Res. 52 W194 W199 10.1093/nar/gkae415 38783102 PMC11223844

[25] Pavesic M.W. Gale A.N. Nickels T.J. Harrington A.A. Bussey M. Cunningham K.W 2024 Calcineurin-dependent contributions to fitness in the opportunistic pathogen *Candida glabrata* mSphere 9 e00554-23 10.1128/msphere.00554-23 38171022 PMC10826367

[26] Juvvadi P.R. Lee S.C. Heitman J. Steinbach W.J 2017 Calcineurin in fungal virulence and drug resistance: prospects for harnessing targeted inhibition of calcineurin for an antifungal therapeutic approach Virulence 8 186 197 10.1080/21505594.2016.1201250 27325145 PMC5354160

[27] Yadav V. Heitman J 2023 Calcineurin: The Achilles’ heel of fungal pathogens PLoS Pathog. 19 e1011445 10.1371/journal.ppat.1011445 37410706 PMC10325075

[28] Zhang Q.Q. Ma M. Hua R. Lu Y. Shi B.R. Wu Q.C et al. 2023 Effect of farnesol on glucan-related genes in Candida albicans biofilm and the drug resistance of Candida albicans Stomatology 43 488 493 10.13591/j.cnki.kqyx.2023.06.002

[29] Borchani C. Fonteyn F. Jamin G. Paquot M. Blecker C. Thonart P 2014 Enzymatic process for the fractionation of baker’s yeast cell wall (Saccharomyces cerevisiae) Food Chem. 163 108 113 10.1016/j.foodchem.2014.04.086 24912704

[30] Gaálová-Radochová B. Kendra S. Jordao L. Kursawe L. Kikhney J. Moter A. et al. 2023 Effect of quorum sensing molecule farnesol on mixed biofilms of Candida albicans and Staphylococcus aureus Antibiotics (Basel). 12 441 10.3390/antibiotics12030441 36978309 PMC10044556

[31] Kovács R. Majoros L 2020 Fungal quorum-sensing molecules: a review of their antifungal effect against *Candida* Biofilms J. Fungi (Basel). 6 99 10.3390/jof6030099 32630687 PMC7559060

[32] Juvvadi P.R. Fox D. 3rd Bobay B.G. Hoy M.J. Gobeil S.M.C. Venters R.A. et al. 2019 Harnessing calcineurin-FK506-FKBP12 crystal structures from invasive fungal pathogens to develop antifungal agents Nat. Commun. 10 4275 10.1038/s41467-019-12199-1 31537789 PMC6753081

[33] Gupta P. Sharma M. Arora N. Pruthi V. Poluri K.M 2018 Chemistry and biology of farnesol and its derivatives: quorum sensing molecules with immense therapeutic potential Curr. Top. Med. Chem. 18 1937 1954 10.2174/1568026619666181210124159 30526460

[34] Pavesic M.W. Gale A.N. Nickels T.J. Harrington A.A. Bussey M. Cunningham K.W 2024 Calcineurin-dependent contributions to fitness in the opportunistic pathogen Candida glabrata mSphere 9 e00554-23 10.1128/msphere.00554-23 38171022 PMC10826367

[35] Van der Does D. Boutrot F. Engelsdorf T. Rhodes J. McKenna J.F. Vernhettes S. et al. 2017 The Arabidopsis leucine-rich repeat receptor kinase MIK2/LRR-KISS connects cell wall integrity sensing, root growth and response to abiotic and biotic stresses PLoS Genet. 13 e1006832 10.1371/journal.pgen.1006832 28604776 PMC5484538

[36] Crump G.M. Rozovsky S. Grimes C.L 2022 Purification and characterization of a stable, membrane-associated peptidoglycan responsive adenylate cyclase LRR domain from human commensal *Candida albicans* Biochemistry 61 2856 2860 10.1021/acs.biochem.2c00305 35816699 PMC9771868

[37] Berkow E.L. Lockhart S.R. Ostrosky-Zeichner L 2020 Antifungal susceptibility testing: current approaches Clin. Microbiol. Rev. 33 e00069-19 10.1128/CMR.00069-19 32349998 PMC7194854

[38] Miyazaki T. Yamauchi S. Inamine T. Nagayoshi Y. Saijo T. Izumikawa K. et al. 2010 Roles of calcineurin and Crz1 in antifungal susceptibility and virulence of Candida glabrata Antimicrob. Agents Chemother. 54 1639 1643 10.1128/AAC.01364-09 20100876 PMC2849377

[39] Cen Y. Fiori A. Van Dijck P 2015 Deletion of the DNA ligase IV gene in Candida glabrata significantly increases gene-targeting efficiency Eukaryotic Cell 14 783 791 10.1128/EC.00281-14 26048009 PMC4519754

[40] Cen Y. Timmermans B. Souffriau B. Thevelein J.M. Van Dijck P 2017 Comparison of genome engineering using the CRISPR-Cas9 system in C. glabrata wild-type and lig4 strains Fungal Genet. Biol. 107 44 50 10.1016/j.fgb.2017.08.004 28822858

[41] Carolus H. Sofras D. Boccarella G. Sephton-Clark P. Biriukov V. Cauldron N.C. et al. 2024 Acquired amphotericin B resistance leads to fitness trade-offs that can be mitigated by compensatory evolution in Candida auris Nat. Microbiol. 9 3304 3320 10.1038/s41564-024-01854-z 39567662

