# Supplementary material for: Targeting calcineurin: a synergistic strategy with caspofungin to induce farnesol-mediated quorum sensing in Nakaseomyces glabrata—an inoculum-dependent tale
Source: Biochem J. 2025 Dec 24;483(1):BCJ20253193. doi: 10.1042/BCJ20253193 (PMC12794322; doi:10.1042/BCJ20253193)
Supplement: online supplementary material 1. [file bcj-483-1-BCJ20253193-s001.docx]

# Supplementary material

**Targeting calcineurin: a synergistic strategy with caspofungin to induce farnesol-mediated quorum sensing in *Nakaseomyces glabrata*—An inoculum-dependent tale**

Yu-Ke Cen, Lu-Lu Zhang, Zi-Jie Zhang, Yu-Jie Zhang, Tao-Xu Lu, Wan-Ying Zhou, Chao Xiang, Ya-Ping Xue^,*^, Yu-Guo Zheng

**Table S1.** **Strains and primers used in this study.**

| Strains | | |
| --- | --- | --- |
| Strain | **Genotype or description** | **Source** |
| 2001HT (WT) | *his3Δtrp1Δ* (CBS138 background strain) | (Miyazaki et al., 2010) |
| *cnb1Δ* | *cnb1Δ* (2001HT background strain) | (Miyazaki et al., 2010) |
| *cnb1Δdpp3Δ* | *cnb1Δdpp3Δ* (*cnb1Δ* background strain) | This study |
| Primers | | |
| Use of primers | **Name** | **Sequence (5’-3’)** |
| Construction of *DPP3* deletion strain | G-a-F | CACATCTTGTTGCAGTCATC |
|  | G-a-R | TTATACTGATCAGTGGGCACT |
|  | G-b-F | ATGCAAGCTGTTACACTAGC |
|  | G-b-R | TGGTTGGGTAGAAAGTATCG |
|  | verification-F | AGTCCTATAACACCACCTG |
|  | verification-R | CGTTAGTATCGAATCGACAGC |
| Real-time PCR analysis. | ACT1-F | CGCTTTGGACTTCGAACAAGAA |
|  | ACT1-R | GTTACCGATGGTGATGACTTGAC |
|  | DPP3-F | TCCCATCCATATGCGGAGGT |
|  | DPP3-R | GGTCGGCAAAAAGAAGCCAA |
|  | ERG11-F | CGGCTCTGCTATCCCATACG |
|  | ERG11-R | ATTCGTGACCCTTTGGACCC |
|  | FKS1-F | ATCAACCACCAGCTGCCTAC |
|  | FKS1-R | GCACCTGCCCATTTTCTTGG |
|  | FKS2-F | CTGGTGTTGGCAATGGGTTG |
|  | FKS2-R | ATTCATACCGTTGCTGCCCA |

**Figures**

**Figure S1**


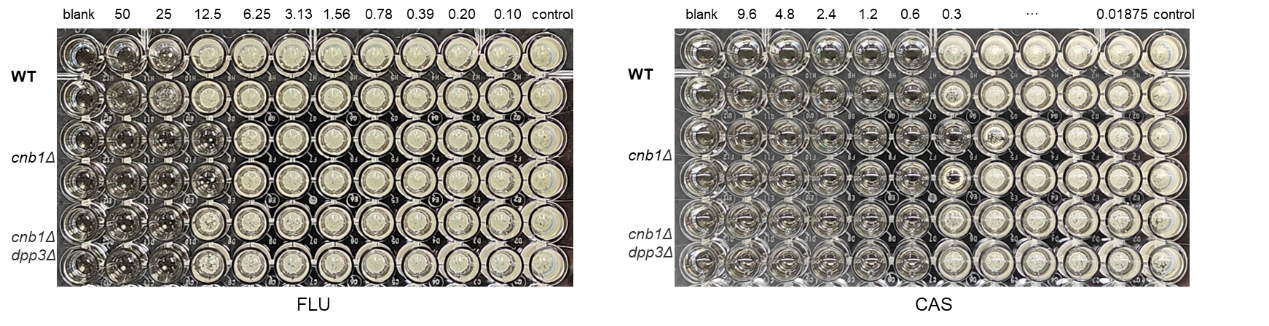


**Figure S1.** **Minimum Inhibitory Concentration** **(MIC) assay for the wild-type (WT), *cnb1Δ*, and *cnb1Δdpp3Δ* strains against fluconazole and caspofungin**. As indicated: row 1, 2: repeats of the WT strain; row 3, 4: repeats of the *cnb1Δ* strain; row 5, 6: repeats of the *cnb1Δdpp3Δ* strain. Lane 1: the negative control without cells added. Lane 12: the positive control without antibiotics added. Lane 2–11: serial 2-fold diluted fluconazole (with concentration from 50 to 0.1 μg/mL) or caspofungin (with concentration from 9.6 to 0.01875 μg/mL). MIC was determined using the broth dilution method according to CLSI standard M27-A4. Freshly grown cells were water diluted to a concentration of 5 × 10^6^ cells/mL. The prepared inoculum was diluted 1,000-fold in YPD medium. The experiment was repeated three times. The plate was incubated at 37°C for 24-36 h before observing the results. FLU: fluconazole; CAS: caspofungin.

**Figure S2**


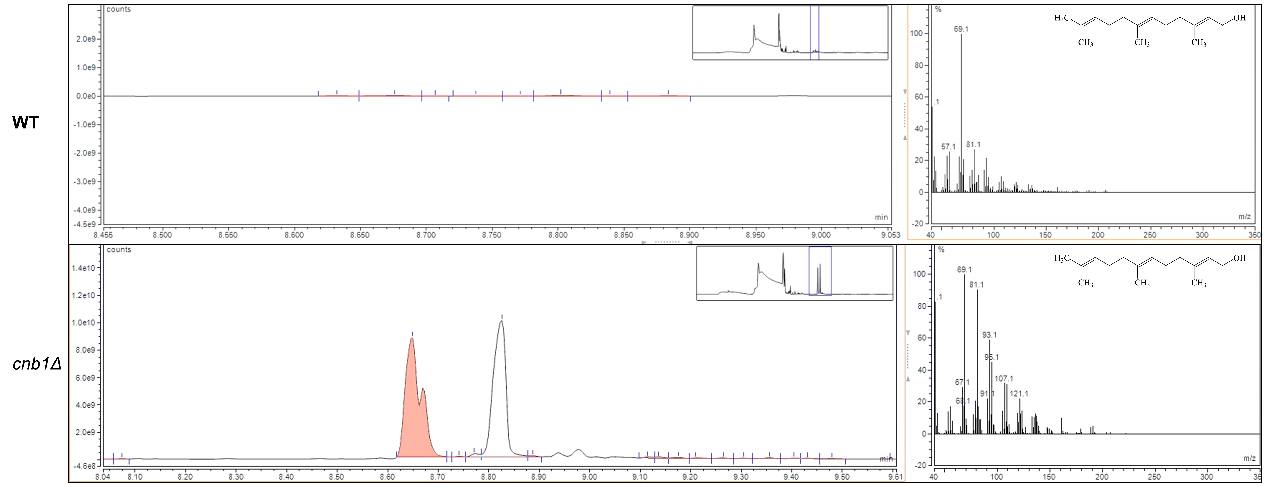


**Figure S2.** **GC-MS analysis of farnesol in the growth medium of the wild type strain and calcineurin mutant (*cnb1Δ*).** Cells were grown for 14 h to reach the logarithmic phase. Caspofungin was subsequently added at a sub-MIC concentration of 0.1 μg/mL, followed by incubation for another 6-8 h. Dodecane (10% of the total volume) was added for extraction. The upper dodecane layer was collected and the farnesol content was determined by GC-MS. The chromatographic conditions: 1 µL sample volume (no split flow) was used, the initial temperature was 100 °C, and raised to 280 °C at the speed of 15 °C /min, and kept at this temperature for 5 min. The determination was implemented by Thermo Scientific™ TRACE™ 1300 equipped with an HP-5 (Agilent J&W HP-5MS 12m, 0.20mm, 0.33um) column. For mass spectrometry, electron bombardment ion (EI) was used as the ion source, and heated to 230℃, with a solvent delay of 5 min, scanning range of 40~400 an, using atlas to retrieve qualitative retention time, and selecting ions 69 and 81 for quantification. The determination was implemented using Thermo Scientific™ ISQ™ 7000.

**Figure S3**


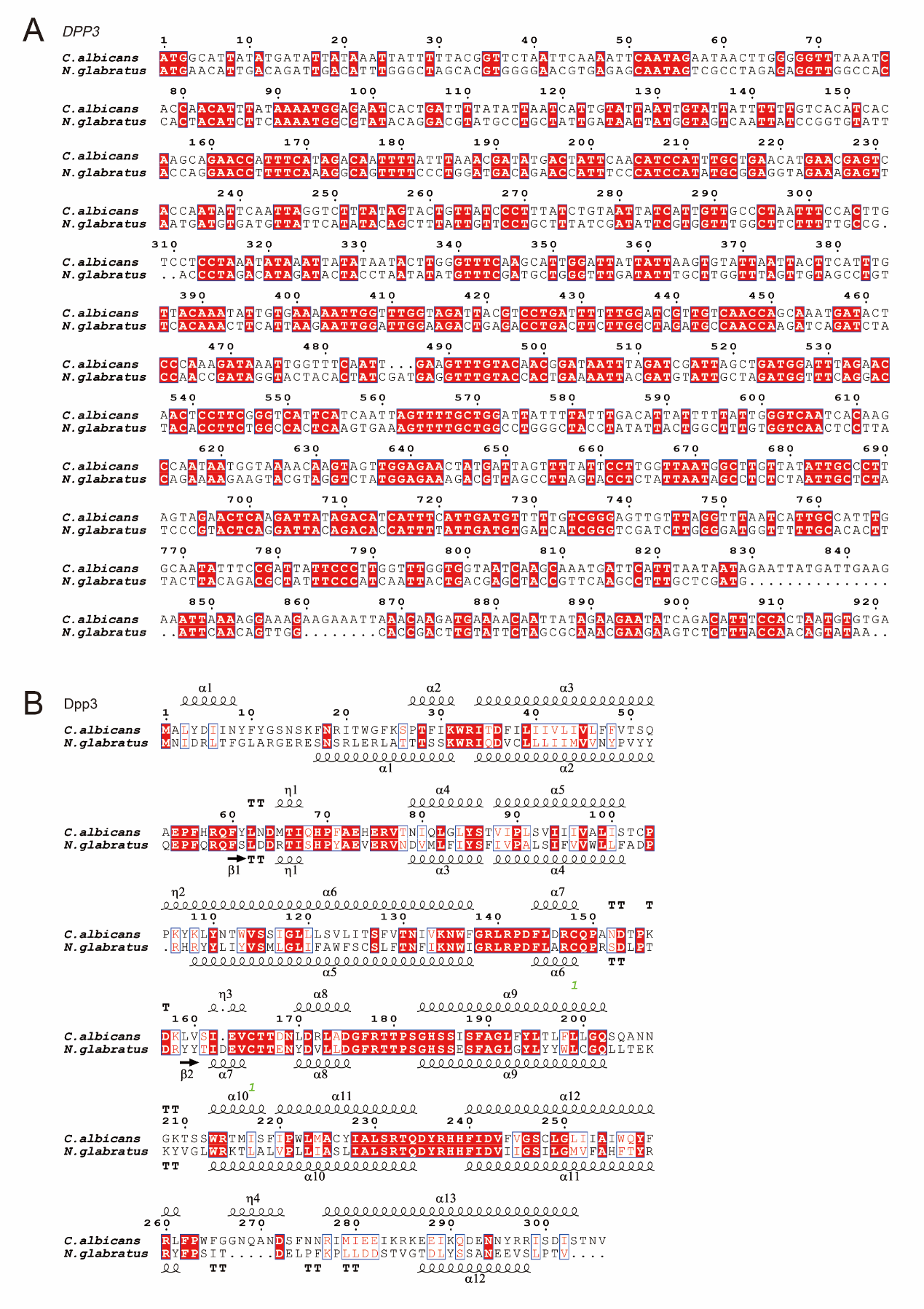


**Figure S3.** **Sequence and structural alignment of *C. albicans* Dpp3 and *N. glabrata* Dpp3. (A):** DNA sequence alignment of *C. albicans* *DPP3* and *N. glabrata* *DPP3*. **(B):** Alignment of protein sequences of *C. albicans* Dpp3 and *N. glabrata* Dpp3. The alignment was first implemented by CLUSTALW. Incorporating the alignment results and the PDB structural information of Dpp3, we generated the current figure using the ESPript website (http://espript.ibcp.fr/ESPript/cgi-bin/ESPript.cgi).

**Figure S4**


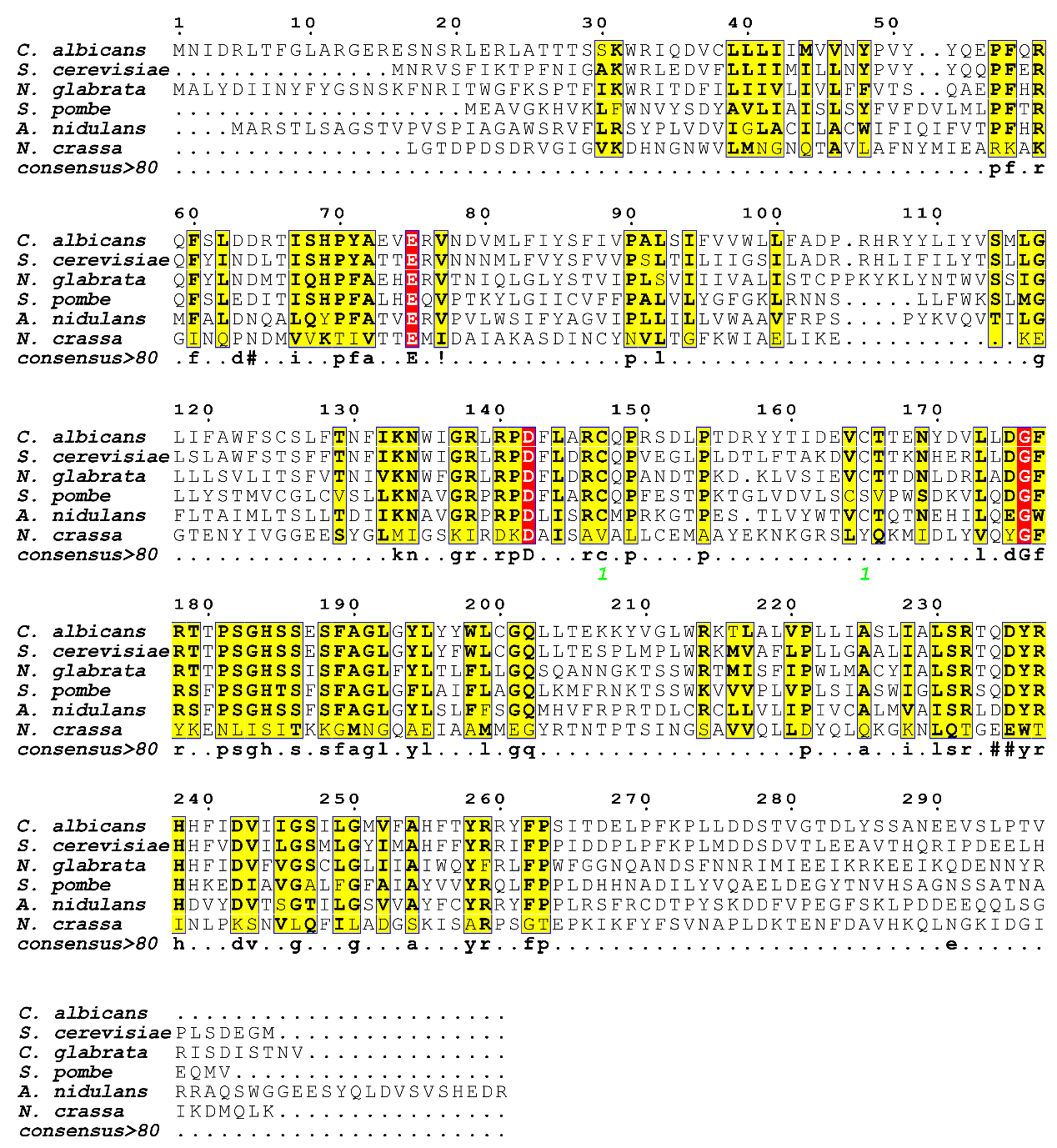


**Figure S4. Cross-genus alignment of the homologous Dpp3 amino acid sequences.** The alignment was performed using Dpp3 homologous proteins from *C. albicans* (C7_02500C_A), *N. glabrata* (CAGL0H01177g), *Aspergillus nidulans* (AN2124), *Neurospora crassa* (NCU05941), *Schizosaccharomyces pombe* (SPBC409.18), and *Saccharomyces cerevisiae* (YDR284C). The conservation analysis was conducted using the ESPript website (https://espript.ibcp.fr/ESPript/ESPript/index.php). Amino acids highlighted in red indicate a 100% consensus rate, while those highlighted in yellow represent an 80% consensus rate.

**Figure S5**


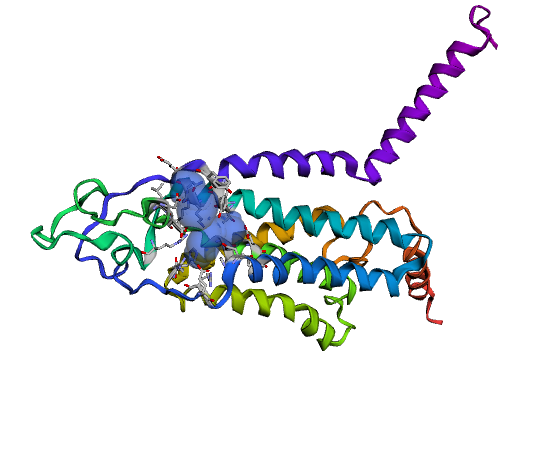


**Figure S5. Predicted maximum cavity of the Dpp3 protein in *N. glabrata* for identifying the catalytic active pocket.** The three-dimensional structure was visualized using PyMol, and the putative catalytic cavity (highlighted as a blue transparent area) was predicted using the CASTpFold web server (https://cfold.bme.uic.edu/castpfold/). Secondary structures are color-coded, with gray and red indicating residues surrounding the predicted cavity.
